# Precision oncology in advanced melanoma: molecular landscapes and personalized therapy recommendations from the Molecular Tumor Board Freiburg

**DOI:** 10.1038/s41698-026-01763-w

**Published:** 2026-10-21

**Authors:** T. Lowinus, V. Schipperges, P. Metzger, T. Brummer, U. Matysiak, E. P. Dopfer, M. E. Hess, M. Fahrner, T. Pauli, A. Blaumeiser, H. Becker, J. Kuehn, M. Rassner, J. Falkenstein, H. Bertemes, P. Bovio, N. Pinter, S. Cysar, S. Lehr, R. Wäsch, L. Gräßel, K. Eyerich, A. M. Schultheis, O. Schilling, S. Lassmann, M. Werner, A. L. Illert, J. Duyster, R. Zeiser, C. C. Miething, M. Boerries, F. Meiss, D. Rafei

**Affiliations:** 1 Department of Medicine I—Medical Centre—University of Freiburg, Faculty of Medicine, University of Freiburg, Freiburg, Germany; 2 Comprehensive Cancer Center Freiburg, Medical Center—University of Freiburg, Faculty of Medicine, University of Freiburg, Freiburg, Germany; 3 Institute of Medical Bioinformatics and Systems Medicine (IBSM), Medical Center—University of Freiburg, Faculty of Medicine, University of Freiburg, Freiburg, Germany; 4 German Cancer Consortium (DKTK), Partner Site Freiburg, a partnership between DKFZ and Medical Center—University of Freiburg, Freiburg, Germany; 5 Institute of Molecular Medicine and Cell Research (IMMZ), Faculty of Medicine, University of Freiburg, Freiburg, Germany; 6 Institute for Surgical Pathology, Medical Center—University of Freiburg, Faculty of Medicine, University of Freiburg, Freiburg, Germany; 7 Department of Dermatology and Venereology—Medical Centre—University of Freiburg, Faculty of Medicine, University of Freiburg, Freiburg, Germany; 8 Department of Hematology and Medical Oncology, University Medical Center Göttingen, Göttingen, Germany; 9 Department of Medicine III, Faculty of Medicine, Klinikum Rechts der Isar, Technical University Munich (TUM), Munich, Germany; 10 Center of Personalized Medicine, Technical University of Munich (TUM), University Hospital, Technical University of Munich, Munich, Germany

**Keywords:** Biomarkers, Cancer, Oncology

## Abstract

Immune checkpoint inhibitors (ICIs) and BRAF/MEK kinase inhibitors have substantially improved survival in advanced melanoma; however, therapeutic resistance and severe toxicities underscore the ongoing need for alternative strategies. Here, we evaluate the clinical utility of multi-omic profiling, biomarker-stratified recommendations, and survival outcomes within a prospective cohort of 98 patients with advanced cutaneous or uveal melanoma evaluated by the Molecular Tumor Board Freiburg (MTB-FR) between 2021 and 2024. Completed profiling revealed at least one potential actionable target beyond classical *BRAF* V600 mutations in 83.8% of cases. Alterations in *NRAS*, atypical *BRAF* variants, *CDK4/6* pathway amplifications, and DNA-damage response (DDR) gene mutations most frequently guided board recommendations. Biomarker-stratified therapies were recommended for 68.4% of patients, and personalized immunotherapy combinations for 72.4%. The treatment implementation rate was 33.8% (n = 27), achieving an overall disease control rate (DCR) of 42.9% and an overall response rate (ORR) of 10.7%. These findings demonstrate that comprehensive molecular profiling in a real-world setting uncovers actionable alterations, providing viable subsequent treatment avenues that may optimize outcomes for heavily pretreated melanoma patients beyond established standards of care. Ultimately, prospective, randomized, biomarker-driven trials remain essential to validate whether these individualized diagnostic strategies translate into a reproducible survival benefit.

## Introduction

Malignant melanoma is the deadliest form of skin cancer, arising from neoplastic melanocytes and characterized by early metastatic potential. Due to changing lifestyle habits and increased exposure to ultraviolet (UV) radiation as the primary risk factor, a significant rise in incidence is projected over the coming decades, substantially increasing the global clinical burden^1–3^. Systemic treatment options for advanced melanoma have expanded significantly in recent years, markedly improving overall survival (OS) for this aggressive malignancy. Immune-checkpoint inhibitors (ICIs) have transformed systemic therapy by enabling durable responses and have consequently become the established first-line treatment for stage IV melanoma^4–6^. However, most patients eventually exhibit primary or secondary resistance to ICI or discontinue therapy due to severe immune-related adverse events (irAEs), ultimately leading to disease progression and mortality^7^. Oncogenic mutations within malignant melanocytes contribute to ICI resistance by promoting an immunosuppressive tumor microenvironment^7–9^. Primary and secondary resistance to ICI are multifaceted processes driven by both tumor-intrinsic and tumor-extrinsic mechanisms. Intrinsic factors include the loss of neoantigen expression, defective antigen presentation via HLA class I and II down-regulation^10^, and mutations in interferon-gamma signaling pathways (e.g., JAK1/JAK2). Extrinsically, the accumulation of immunosuppressive cell types, such as regulatory T cells (T_regs_), myeloid-derived suppressor cells, and tumor-associated macrophages, alongside the upregulation of alternative immune checkpoints (e.g., LAG-3, TIM-3) heavily restricts T-cell-mediated antitumor immunity^11–18^.

Because mitogen-activated protein kinase (MAPK) signaling critically regulates the survival, proliferation, angiogenesis, and migration of melanocytes^19^, BRAF/MEK kinase inhibitors can prolong progression-free survival in patients with *BRAF* V600-mutated melanoma. Nevertheless, secondary drug resistance frequently emerges^20^, typically mediated through reactivation of the MAPK pathway via *NRAS* mutations, *BRAF* V600E amplification, or alternative splicing variants, as well as parallel pathway activation such as the PI3K/AKT/mTOR axis^21–23^. Moreover, BRAF/MEK inhibition is not a viable option for *BRAF* V600 wild-type disease, which constitutes approximately 50–60% of all melanoma cases^24^. Given this intricate landscape of interconnected, multi-clonal resistance mechanisms^25^, standardized subsequent treatment lines often fail, underlining the clinical relevance of comprehensive high-throughput multi-omic characterization to uncover actionable non-hotspot alterations.

Consequently, there is an ongoing translational requirement to elucidate these multi-layered resistance mechanisms and identify alternative molecular targets. With the emergence of high-throughput sequencing and multi-omic technologies, characterization of the molecular landscape of melanoma has advanced considerably^26–29^. To date, however, *BRAF* V600 mutations remain the only oncogenic alterations with regulatory approval for targeted systemic treatment in melanoma^19^. The central challenge remains translating the identification of tumorigenic drivers and oncogenic variants into tangible clinical benefits for patients^30^. Recent efforts in molecularly guided, personalized clinical decision-making have demonstrated encouraging initial results across various solid tumors, including melanoma^31–33^. However, real-world cohort data evaluating the systematic integration of multi-omic profiling specifically for heavily pretreated cutaneous and uveal melanoma patients remain sparse.

Molecular tumor boards (MTBs) serve as established interdisciplinary platforms that facilitate individualized molecular tumor profiling and provide personalized therapy recommendations for patients experiencing disease progression beyond the standard of care. The MTB of the Comprehensive Cancer Center Freiburg (MTB-FR), Germany, utilizes a comprehensive portfolio of multi-omic analyses and supportive biomarker assays of tumor specimens, followed by interdisciplinary case discussions to recommend biomarker-informed treatments within a prospective observational study framework^34,35^ (DRKS00025847). Here, we evaluate the clinical utility of this approach by reporting the individual molecular profiles, the resulting therapeutic recommendations, the subsequent implementation rates of biomarker-stratified and personalized therapies, and the clinical outcomes of 98 patients with advanced cutaneous or uveal melanoma evaluated by the MTB-FR between January 2021 and December 2024.

## Results

### Patient characteristics

**Fig. 1:**
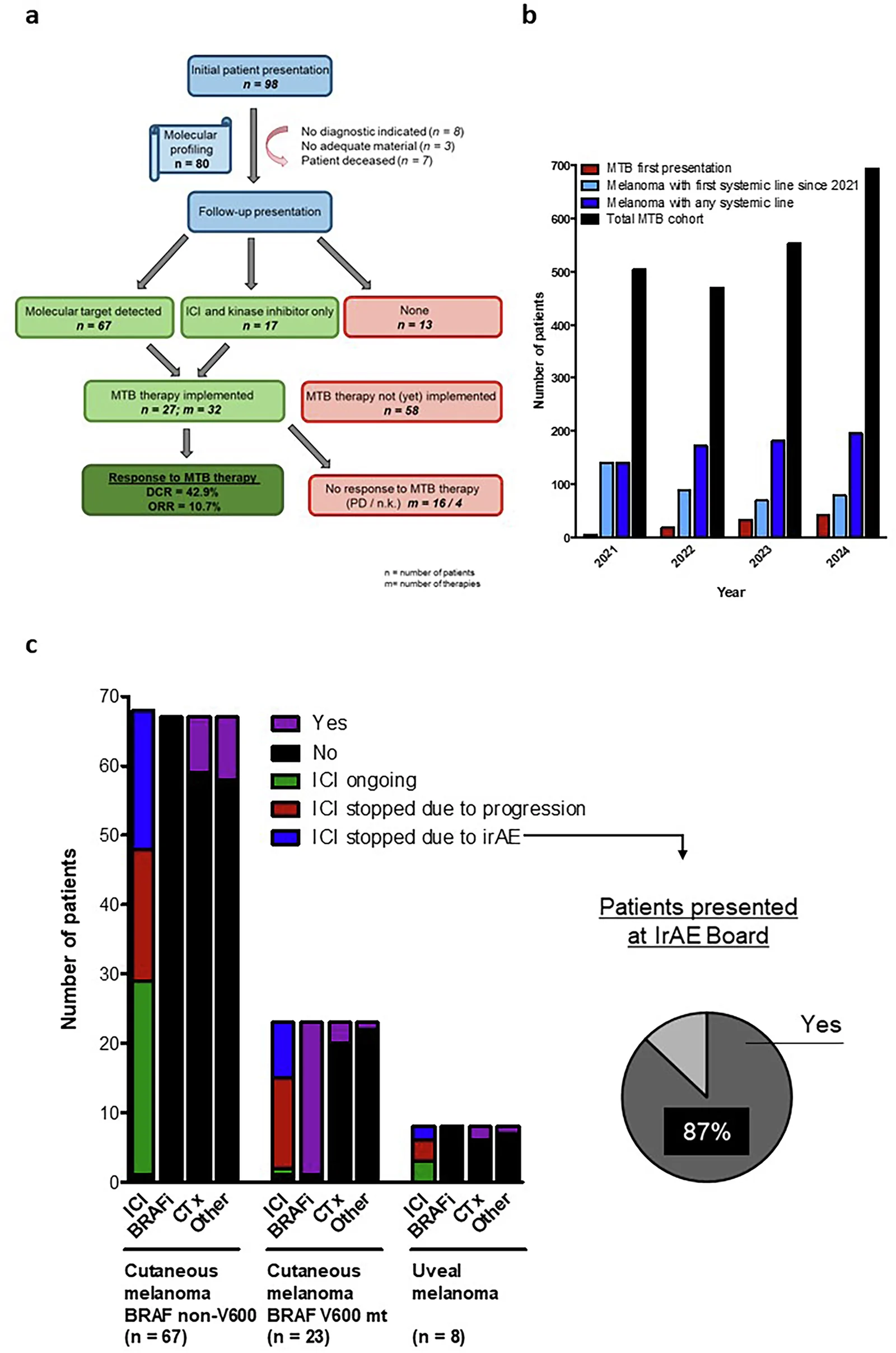
The workflow delineates enrollment pathways within the MTB-FR melanoma cohort, while integrated graphs display cohort-specific patient characteristics. **a** Overview of the MTB-FR melanoma cohort pipeline from initial case registration and multi-omic profiling to interdisciplinary follow-up presentation, formulation of treatment recommendations, clinical implementation, and efficacy outcomes. **b** Annual number of melanoma patients discussed at the MTB-FR (red bars), melanoma patients receiving the first (bright blue bars) or any (blue bars) systemic therapy line at the Cancer Center Freiburg, and the total pan-cancer MTB cohort (black bars) from 2021 to 2024. **c** Patient distribution within the melanoma cohort (total *n* = 98) stratified by disease biology into BRAF wild-type (non-V600-mutant) cutaneous melanoma, BRAF V600-mutant cutaneous melanoma, and uveal melanoma. For each subgroup, pre-MTB treatment status indicates patients exposed to specified therapies prior to enrollment. Immune-checkpoint inhibitor (ICI) status prior to MTB enrollment is categorized into ongoing ICI treatment due to clinical response (green bar sections), ICI discontinued due to melanoma progression (red bar sections), or ICI discontinued due to immune-related adverse events (irAEs, blue bar sections). Exposure to BRAF/MEK inhibition, chemotherapy, or other treatments prior to MTB enrollment is indicated as yes (violet bar sections) or no (black bar sections). The inset pie chart displays the percentage of patients concurrently evaluated by the institutional multidisciplinary irAE board due to severe (grade 3–4) or rare toxicities. BRAFi BRAF inhibitor, CR complete response, CTx chemotherapy, DCR disease control rate (CR + PR + SD), ICI immune checkpoint inhibitor, irAE immune-related adverse event, m therapy count, MTB-FR Molecular Tumor Board Freiburg, *n* patient count, n.k. not known, ORR overall response rate (CR + PR), PR partial response, SD stable disease. Other treatments include interleukin-2, interferon, and tebentafusp.

Between January 2021 and December 2024, a total of 98 patients with advanced melanoma treated at the University Medical Center Freiburg were registered and evaluated within the MTB-FR cohort analysis. To track the longitudinal clinical utility of this approach, Fig. 1a outlines the comprehensive patient journey from initial presentation and molecular profiling through to the formulation, implementation, and clinical efficacy of personalized treatment recommendations.

To contextualize this subcohort within the institutional oncology volume, these 98 individuals represented 4.4% of the entire, pan-cancer cohort (*N* = 2221) evaluated by the MTB-FR during this 4-year period. Furthermore, when compared to the total population of advanced melanoma patients (with regional lymph node or distant metastases) treated with any systemic therapy at our cancer center during this timeframe, the MTB-FR cohort represents a selective subgroup of 26.0%. During this period, the annual number of presented melanoma patients increased from 5 patients in 2021 to 42 patients in 2024, reflecting increased utilization of molecular profiling. Concurrently, from 2021 to 2024, the percentage of melanoma patients discussed in the MTB out of all those receiving first-line systemic therapy increased from 3.5 to 53.2% (Fig. 1b).

Cutaneous melanoma was diagnosed in 92% of the cohort, comprising 68% with *BRAF* V600 wild-type disease and 24% harboring a *BRAF* V600 mutation, whereas the remaining 8% of patients presented with uveal melanoma (Fig. 1c). At the time of initial case presentation to the MTB-FR, the baseline characteristics were as follows: the median age of the cohort was 62 years, with an overall range spanning 20–92 years (Fig. S1a). The majority of patients (91%) presented with distant metastatic disease classified as AJCC stage IV^36^, including 41% with confirmed brain metastases, whereas a minority (9%) had locally advanced disease categorized as AJCC stage III^36^ (Fig. S1b, c).

All patients had received prior systemic therapy. Referral to the MTB-FR required documented disease progression, intolerance to standard-of-care options with no remaining conventional treatment avenues, or the anticipation thereof as determined by the treating physician. Except for two patients (2.0%), who were excluded from standard frontline protocols due to either a severe pre-existing autoimmune disorder or a poor Eastern Cooperative Oncology Group performance status, the majority of patients had been exposed to ICI prior to MTB registration, including 85.7% with dual ICI and 12.3% with mono-ICI (Fig. 1c). At the time of MTB registration, approximately one-third of the patients were actively continuing ICI due to an ongoing adequate clinical response, specifically complete remission (CR), partial remission (PR), or stable disease (SD), whereas the remaining two-thirds had discontinued ICI due to either disease progression or severe (grade 3–4) irAEs. To support side-effect management, 87% of patients experiencing severe toxicities were concurrently evaluated by the institutional multidisciplinary irAE board prior to formal ICI cessation (Fig. 1c). Furthermore, 21 out of 23 patients (91.3%) harboring a *BRAF* V600 mutation had received BRAF/MEK inhibitor therapy prior to MTB referral. In one of the remaining two patients, the *BRAF* V600 mutation had been identified with a low variant allele frequency (VAF) of 5.3% in a liver metastasis (with 30% histological tumor cell content); the second patient initiated targeted therapy promptly following MTB evaluation. Additional lines of pre-MTB systemic treatment included dacarbazine-based chemotherapy, intralesional interleukin-2, or the gp100-directed T cell receptor therapeutic tebentafusp for uveal melanoma (Fig. 1c). Overall, patients had been treated with a mean of 1.5 (range: 0–4) systemic therapeutic lines prior to MTB-FR registration (Fig. S1d).

### Molecular tumor profiling

Tumor molecular profiling was performed and evaluated in 80 out of 98 initially registered patients (81.6%). In the remaining 18 cases, molecular diagnostics were either not requested by the treating physician (*n* = 8), were not feasible due to inadequate tumor tissue quantity or quality (*n* = 3), or could not be completed because patients passed away during the diagnostic workflow (*n* = 7; Fig. 1a). Specifically, the reasons for intentionally forgoing comprehensive molecular sequencing included the prior detection of an actionable driver (e.g., an atypical BRAF variant) during routine diagnostics, rendering further time-consuming panel sequencing clinically redundant. Alternatively, a rapid decline in general performance status necessitated immediate therapeutic recommendations from the board based on existing records, without delaying clinical care for the duration of a full sequencing workflow.

**Fig. 2:**
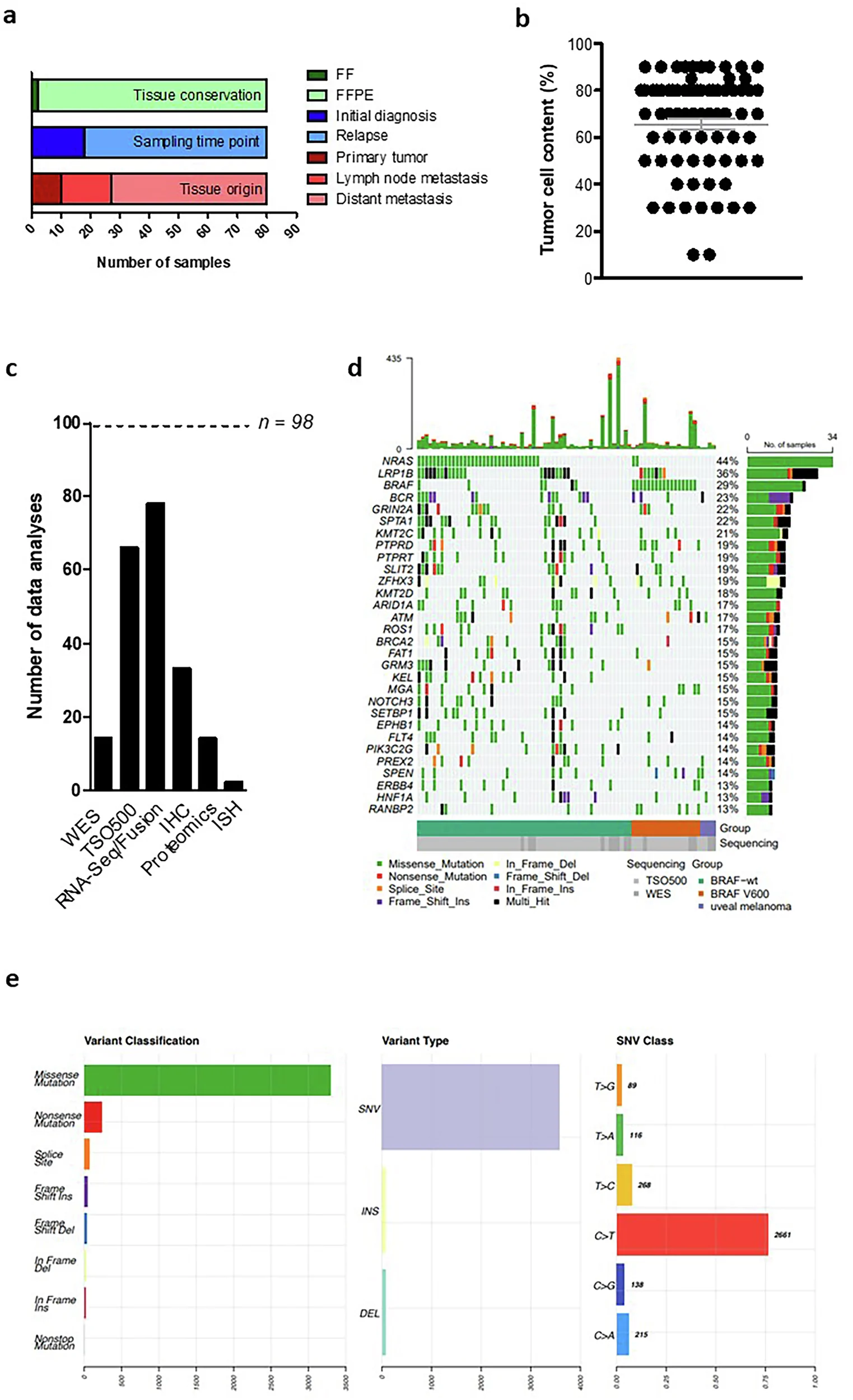
Comprehensive molecular profiling outlines the genomic landscape of the MTB-FR melanoma cohort. **a** Visual representation of tumor tissue specifications utilized for molecular profiling. Upper bar shows the proportion of formalin-fixed paraffin-embedded tissue (FFPE) versus fresh-frozen tissue (FF); middle bar shows the proportion of tissue obtained at initial diagnosis versus disease relapse; lower bar shows the proportion of tissue derived from the primary tumor, lymph node metastases, or distant metastases. **b** Dot plots depicting the histopathological tumor cell content within profiled specimens, where horizontal lines indicate the mean and error bars represent the standard error of the mean (SEM). **c** Methodological breakdown of the tumor profiling pipeline, showing the absolute number of patient samples analyzed per modality. **d** OncoPrint diagram presenting the somatic mutational landscape of 78 successfully genotyped melanoma patients via WES or the TSO500 assay, grouped into three cohorts: BRAF wild-type (non-V600-mutant) cutaneous melanoma, BRAF V600-mutant cutaneous melanoma, and uveal melanoma. The 30 most frequently altered genes within the overall cohort are displayed. Each column represents an individual tumor specimen, and each row corresponds to a specific gene. The percentage of altered cases per gene is depicted as a bar graph on the right. Individual sequencing modalities, clinical subgroups, and tumor mutational burden (TMB) values are aligned vertically. **e** Frequency distribution of variant classifications (left), variant types (middle), and single-nucleotide variant (SNV) mutation classes (right), highlighting the cytosine-to-thymine (C > T) transition predominance. Del deletion, FF fresh-frozen, FFPE formalin-fixed paraffin-embedded, IHC immunohistochemistry, Ins insertion, ISH in situ hybridization, NGS next-generation sequencing, RNA-Seq RNA sequencing, SEM standard error of the mean, SNV single-nucleotide variant, TMB tumor mutational burden, TSO500 TruSight Oncology 500, WES whole-exome sequencing, wt wild-type.

Of the 80 tumor specimens utilized for molecular profiling, with exactly one distinct specimen analyzed per patient (*n* = 80), 66.25% (*n* = 53) originated from distant metastases, 21.25% (*n* = 17) from lymph node metastases, and 12.50% (*n* = 10) from primary tumors. Tissue from disease relapse was used in 77.5% of cases, whereas 22.5% of analyses utilized tissue obtained at initial diagnosis. The majority of profiled samples were formalin-fixed and paraffin-embedded (FFPE; 97.5%), while a minority comprised fresh-frozen tissue (FF; 2.5%; Fig. 2a). The mean histopathological tumor cell content was 65.6% (range: 10–90%; Fig. 2b). Specimens with a tumor cell content below 10% were considered ineligible and were excluded from molecular analyses.

Whole-exome sequencing (WES) was performed in 17.5% (*n* = 14) of analyzed cases, whereas large targeted next-generation sequencing (NGS; Illumina TruSight Oncology 500 [TSO500]) was conducted in 82.5% (*n* = 66). In 97.5% (*n* = 78) of cases, a separate large targeted NGS RNA fusion panel or bulk RNA-sequencing (RNA-Seq) was additionally utilized to detect aberrant gene expression or actionable driver gene fusions, such as those involving *BRAF*, *NTRK*, or *RET* (Fig. 2c).

The genomic landscape is visualized via an OncoPrint diagram, displaying genomic data for 78 of the 80 successfully profiled patients categorized into three cohorts: *BRAF* non-V600 cutaneous melanoma, *BRAF* V600-mutant cutaneous melanoma, and uveal melanoma (Fig. 2d). Two patients were omitted from this visualization due to suboptimal internal quality metrics. For each patient, the 30 most frequently mutated genes, their respective mutation frequencies, the types of alterations (missense, nonsense, insertion, deletion, and frameshift), and the tumor mutational burden (TMB) are displayed. *NRAS* (44%), *BRAF* (29%), *BCR* (23%), *ARID1A* (17%), *ATM* (17%), *ROS1* (17%), *BRCA2* (15%), *NOTCH3* (15%), and *SETBP1* (15%) ranked as the most frequently altered oncogenes. Missense mutations were the predominant aberration type, driven primarily by single-nucleotide variants (SNVs) characterized by a cytosine-to-thymine (C > T) transition (Fig. 2e), a classic signature associated with UV light exposure^37,38^. To evaluate this etiology, a COSMIC mutational signature analysis was performed within our WES subcohort (*n* = 14) only, as the TSO500 assay captures an inherently restricted genomic space, providing insufficient mutational breadth for reliable signature deconvolution. Here, the relative SBS7 activity contribution varied widely across individual patients, spanning values from 0 to 0.903, with a mean contribution of 0.397 (±standard deviation [*s*]: 0.381) and a median contribution of 0.402. Notably, half of the analyzed cutaneous cases (*n* = 7) demonstrated a dominant mutational etiology, with SBS7 accounting for the majority (>0.50) of the total signature exposure (Fig. S2a).

While the preceding analysis focuses exclusively on frequently altered oncogenes, the tumor suppressor gene *LRP1B* emerged as the second most frequently mutated gene overall (36% of cases; Fig. 2d), which is consistent with data from independent melanoma cohorts^39^. *LRP1B* encodes low-density lipoprotein receptor-related protein 1B, and pathogenic mutations in *LRP1B* have been associated with favorable clinical outcomes during ICI therapy across several malignancies, including melanoma^40^. In our selected cohort, 19.2% of patients carried an *LRP1B* mutation classified as pathogenic or likely pathogenic according to a REVEL score ≥0.5^41^; the remaining *LRP1B* variants were classified as benign or variants of unknown significance. Patients harboring pathogenic or likely pathogenic *LRP1B* mutations (*LRP1B*-mt) demonstrated a numerical trend toward a lower rate of primary ICI resistance (20.0% versus 25.4%), but a higher rate of dose-limiting irAEs (40.0% versus 28.6%). The percentage of ICI responders was comparable between the two groups, with 40.0% in the *LRP1B*-mt subgroup versus 42.9% in the *LRP1B* wild-type (*LRP1B*-wt) population (Fig. S2b). To further characterize this major genomic alteration, we evaluated its relationship with the TMB. While *LRP1B* mutations are classically benchmarked as indicators of high genome-wide mutational loads^39,42^, a formal correlation analysis within our cohort revealed a statistically significant elevation of TMB values in the LRP1B-mutated samples (mean: 18.54 vs. 8.31 mut/Mb; *p* < 0.001 via a Mann–Whitney *U* test).

Within the group of cutaneous melanomas without classical *BRAF* V600 mutations (conventionally classified as *BRAF* wild-type), we uncovered atypical non-V600 *BRAF* mutations in 5 out of 58 patients. Together with 4 patients whose atypical *BRAF* mutations had been identified during prior routine diagnostics and who did not undergo repeated in-house sequencing, our cohort exhibited a frequency of 13.4% (9 out of 67 patients) harboring atypical *BRAF* alterations (Fig. S2c), a proportion slightly higher than observed in unselected populations^43,44^. Additionally, two *BRAF* gene fusions were identified via RNA sequencing within this subcohort, bringing the total number of atypical *BRAF* aberrations to 11 (Fig. S2c). This illustrates that in patients previously managed as *BRAF* wild-type, comprehensive multi-omic profiling can uncover atypical *BRAF* aberrations that serve as potential oncogenic drivers and personalized therapeutic targets. Notably, within the group of cutaneous melanomas with classical *BRAF* V600 mutations, one patient with a previously documented *BRAF* V600 mutation (VAF 5.3% in a liver metastasis) showed no detectable mutation in MTB profiling. This discrepancy might be attributed to the low tumor cell content (10%) and/or spatial-temporal tumor heterogeneity during disease progression. For the remaining 5 registered patients with known *BRAF* V600 mutations, adequate material for sequencing was lacking. In contrast, 17 patients had their classical *BRAF* V600 mutation successfully confirmed by sequencing and are displayed in the OncoPrint (Fig. 2d)

The frequency of *NRAS* Q61 mutations was 57.1% in *BRAF* wild-type disease and 11.1% in *BRAF* V600 mutant melanomas (Fig. 2d). While *BRAF* and *NRAS* alterations are typically mutually exclusive in treatment-naïve settings^45,46^, this elevated co-mutation rate corresponds to exactly two patients in our pre-treated collective suggesting the development of secondary *NRAS* variants as a therapeutic escape mechanism^47^. Both individuals had experienced disease progression under prior targeted BRAF/MEK inhibition. Sequencing of the progressive lesion revealed a classical *BRAF* V600E mutation alongside an acquired *NRAS* p.Q61R/K variant (VAF: 48.5%; VAF: 28.1%, respectively), providing a clinical illustration of selection pressure driving polyclonal resistance.

Within the uveal melanoma subgroup, the most frequently mutated gene was GNAQ (75.0% of samples); however, due to the small sample size of this subgroup, GNAQ did not rank among the top 30 altered genes of the overall cohort.

In melanoma, a high TMB, conventionally defined by a threshold of ≥ 10 mutations per megabase (mut/Mb), is associated with enhanced clinical response to ICI therapy^48,49^. This identical threshold was applied uniformly across both sequencing modalities within our pipeline. In our MTB-FR melanoma cohort, we observed a variable TMB, ranging from 0.03 to 99.0 mut/Mb, with a mean of 13.4 mut/Mb and a median of 9.7 mut/Mb. The mean TMB in the *BRAF* non-V600 subgroup (15.6 mut/Mb) was comparable to that of *BRAF* V600 mutant melanoma (10.4 mut/Mb), whereas uveal melanoma exhibited a significantly lower mean TMB of 1.88 mut/Mb (*p* = 0.007 via a Mann–Whitney *U* test), consistent with established literature^50,51^ (Fig. S2d).

Beyond SNVs, we profiled the cohort for copy number alterations (CNAs). Structural variant analysis revealed highly frequent copy number amplifications or gains in *CCND*, *CCNE*, *CDK4*, *CDK6*, and *FGF*(*R*); conversely, *CDKN2A* losses unexpectedly fell outside the top 50 most frequent CNAs (Fig. S2e). To bypass the inherent technical limitations of WES- and panel-based (TSO500) sequencing in robustly detecting copy number losses, particularly for smaller loci like CDKN2A, and to confidently capture functional pathway alterations for MTB recommendations, we integrated orthogonal, protein-level diagnostics, specifically liquid chromatography–mass spectrometry (LC-MS) and immunohistochemistry (IHC), to directly quantify target protein abundance.

### Protein biomarker analyses

**Fig. 3:**
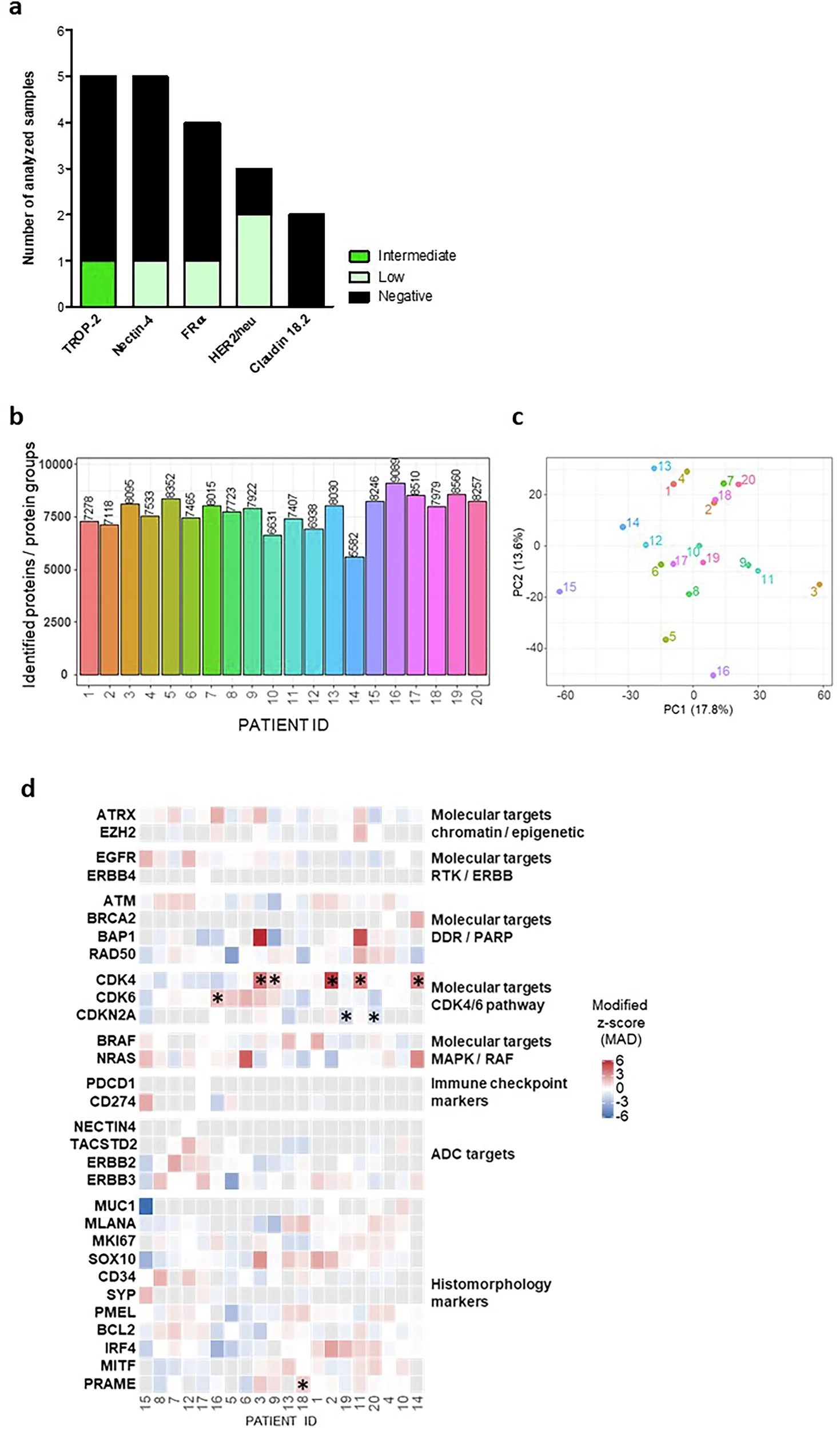
Combined immunohistochemistry (IHC) and liquid chromatography–mass spectrometry (LC-MS) reveal the proteomic landscape of the MTB-FR melanoma cohort. **a** Semiquantitative immunohistochemistry (IHC) profiling of novel antibody–drug conjugate (ADC) targets in selected patient tissue specimens. Staining patterns are categorized into negative (no expression; gray), low expression (H-score 1+ for HER2, H-score of 10–40 for Nectin-4, or H-score 1+ for FRα; bright green), and intermediate expression (H-score of 100–200 for TROP-2; green). TROP-2 and Nectin-4 were evaluated in *n* = 5 patients each, folate receptor alpha (FRα) in *n* = 4 patients, HER2/neu in *n* = 3 patients, and Claudin 18.2 in *n* = 2 patients. **b** Bar plot showing the robust proteome coverage, expressed as the absolute number of uniquely identified proteins or protein groups quantified per patient sample. **c** Unsupervised principal component analysis (PCA) of global protein abundance profiles across the cohort. Each data point represents an individual patient specimen; percentages along the axes indicate the proportion of total variance explained by principal component 1 (PC1) and principal component 2 (PC2). **d** Unsupervised hierarchical clustering and heatmap visualizing molecular target abundance levels, including ADC-associated surface receptors and immune-checkpoint markers, alongside histomorphological differentiation proteins across the screening collective. Expression values are displayed as modified *z*-scores derived from the median absolute deviation (MAD). Protein marker selection was benchmarked against the Pathology Outlines: Invasive Melanoma diagnostic framework. Patient profiles wherein exploratory proteomic data directly informed the final consensus MTB treatment recommendation are annotated with asterisks. ADC antibody–drug conjugate, CDK4 cyclin-dependent kinase 4, DDR DNA damage response, ERBB erb-b receptor tyrosine kinase, FRα folate receptor alpha, HER2 human epidermal growth factor receptor 2, IHC immunohistochemistry, MAD median absolute deviation, MAPK mitogen-activated protein kinase, MTB molecular tumor board, *n* patient count, PARP poly(ADP-ribose) polymerase, PC1 principal component 1, PC2 principal component 2, PCA principal component analysis, RAF rapidly accelerated fibrosarcoma, RTK receptor tyrosine kinase, TROP-2 trophoblast cell-surface antigen 2.

To complement the sequencing data and detect additional therapeutic targets in selected cases, we expanded the diagnostic portfolio to include IHC (33.7%), mass spectrometry-based proteomics (14.3%), or in situ hybridization (ISH; 2.0%; Fig. 2c). Within this portfolio, IHC for programmed death ligand-1 (PD-L1) was routinely performed whenever historical baseline data from initial routine diagnostics were missing. No therapeutic recommendations were based on the PD-L1 IHC results, as all eligible patients had been exposed to ICI prior to MTB referral. In contrast, advanced IHC profiling for the novel antibody–drug conjugate (ADC) targets TROP-2, Nectin-4, HER2, folate receptor alpha (FRα), and Claudin-18.2 was selectively commissioned only for an ultraselected subcohort of six patients, who met three clinical criteria: (1) the definitive clinical exhaustion of all established conventional standard-of-care lines (excluding patients with merely anticipated progression in the near future), (2) a general performance status and comorbidity profile that would clinically permit the administration of an ADC-directed therapeutic protocol, and (3) a lack of high-level actionable genomic driver mutations identified via sequencing. Among the patients evaluated for ADC targets, we identified one case (1 out of 5 tested) with intermediate positive expression for TROP-2, which led to a therapeutic recommendation for the TROP-2-directed ADC sacituzumab govitecan. All remaining analyses for TROP-2, Nectin-4, FRα, HER2, and Claudin-18.2 demonstrated low or negative expression (Figs. 3a and S3a).

LC-MS-based proteomics was incorporated as an exploratory, antibody-independent screening modality to complement genomic profiling and targeted IHC. The combined LC-MS/IHC strategy functioned as a complementary framework, as LC-MS sensitively identified proteins of potential therapeutic relevance, including ADC-associated surface targets, whereas IHC provided tumor-cell-specific and spatially resolved validation in situ. This distinction is critical, as MS-based proteomics cannot inherently localize the detected signal to malignant cells or specific tissue compartments. Conversely, IHC workflows depend heavily on antibody performance, epitope accessibility, and the specific protein region recognized by the diagnostic clone. Consequently, antibody-independent MS-based proteomics serves as a comprehensive scouting approach, while IHC remains indispensable for confirming therapeutically actionable expression and architectural compartmentalization within the relevant tumor niche^52^.

For instance, in one patient within our cohort, LC-MS-based proteomics detected both TROP-2 and Nectin-4 expression, of which only Nectin-4 demonstrated a weak but tumor-cell-specific signal in the subsequent validation IHC staining (Fig. S3b). This case illustrates the potential clinical value of proteomics as a comprehensive screening layer, followed by IHC-based spatial adjudication before therapeutic implementation. As another complementary application, LC-MS-based proteomics provides an orthogonal readout by directly quantifying protein abundance corresponding to copy number variations (CNVs) detected via genomic sequencing. Because CNVs frequently fail to predict actual protein abundance across various malignancies^53^, we profiled CNV-implicated targets. Here, we observed a marked overabundance of the CDK4 protein in a patient harboring a *CDK4* amplification, thereby supporting the clinical rationale for CDK4/6 inhibition. To contextualize this finding, normalized CDK4 protein levels in the MTB patient were benchmarked against a published melanoma proteomic reference cohort^54^ (Fig. S3c).

To provide a broader overview of the proteomic landscape in our MTB-FR melanoma cohort, we evaluated normalized protein abundance profiles from all 20 successfully profiled patients. In the majority of samples, more than 7000 proteins or protein groups were identified and quantified from FFPE tissue, demonstrating robust proteomic coverage in clinically accessible archival specimens (Fig. 3b). Unsupervised principal component analysis (PCA) of global protein abundance profiles did not reveal clearly separated clusters, reflecting the pronounced inter-patient heterogeneity within this heavily selected melanoma population (Fig. 3c). In addition, unsupervised hierarchical clustering of selected proteins visualized therapeutically relevant molecular target classes, including ADC-associated targets, immune-checkpoint markers, and histomorphological melanoma-associated differentiation markers. Samples wherein proteomic profiling informed or supported the final MTB treatment recommendation are annotated with asterisks (*). Notably, the formulation of these clinical recommendations depended not only on the specific molecular event itself, but also on concurrent mutations and the patient’s clinical status (Fig. 3d).

### Molecular targets and treatment recommendations

**Fig. 4:**
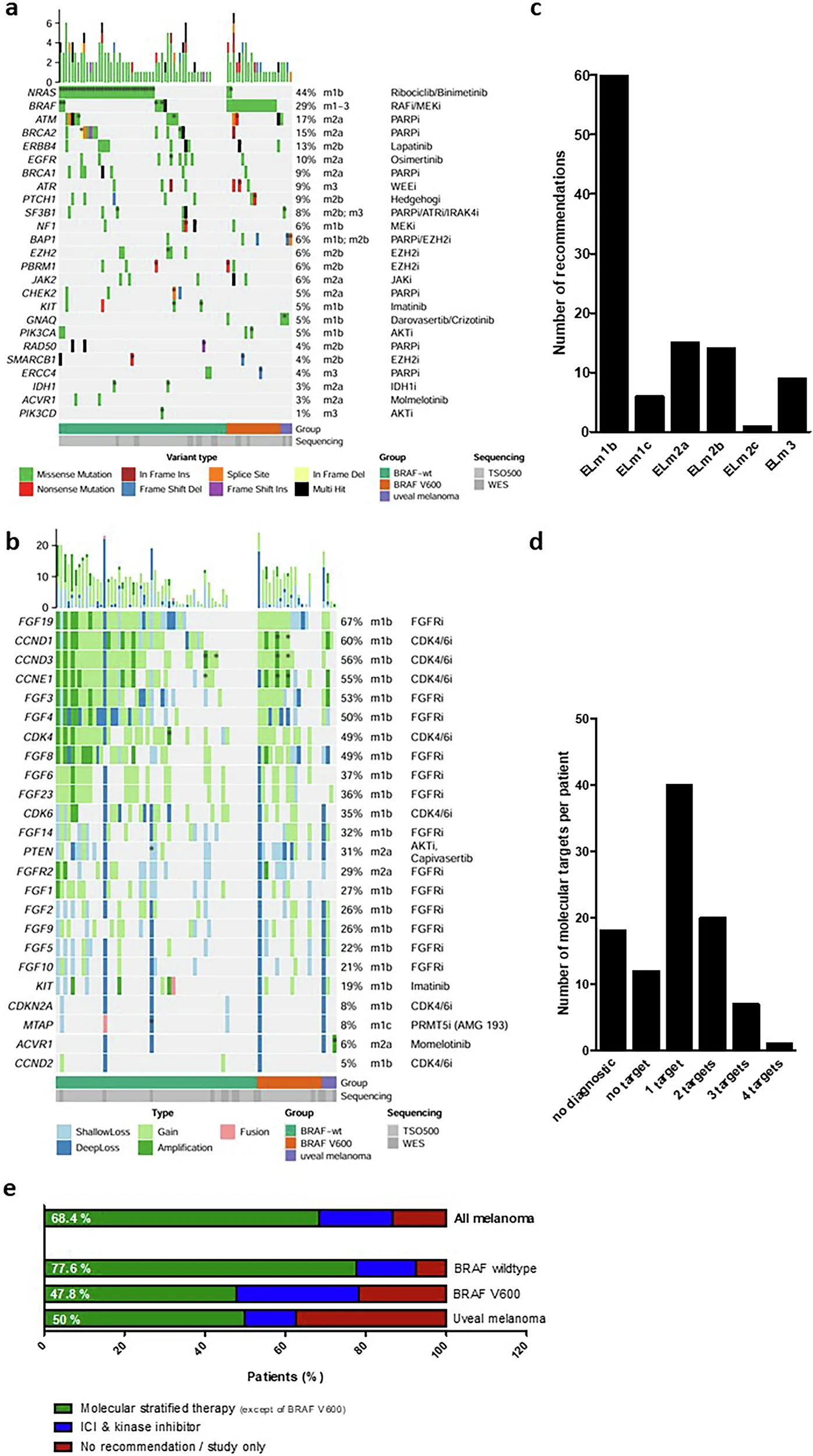
Actionable molecular targets characterize clinical decision-making and inform individualized therapeutic recommendations. Comprehensive overview of the clinical actionability landscape across molecularly profiled patients (*n* = 78) harboring distinct genomic alterations (**a**) and copy number alterations (CNAs, **b**) that directly guided a personalized MTB-FR treatment recommendation. The right-hand annotation matrix integrates the cumulative mutation frequency per gene, the achieved NCT evidence level (ELm1–ELm3), and the specific recommended targeted drugs for each individual gene. Genetic aberrations that informed the final consensus MTB treatment recommendation are annotated with asterisks. **c** Absolute number of biomarker-stratified treatment recommendations categorized according to the nationally established National Center for Tumor Diseases (NCT) precision oncology evidence level framework. **d** Proportion of molecularly profiled patients presenting with zero, one, two, three, or four co-occurring druggable molecular targets utilized for therapeutic decision-making. **e** Overview of percentage-based therapeutic recommendations formulated for the entire melanoma cohort (upper bar) and stratified by clinical subgroups into BRAF wild-type cutaneous melanoma, BRAF V600-mutant cutaneous melanoma, and uveal melanoma. Green bar sections indicate the proportion of patients with at least one molecularly stratified therapeutic recommendation (excluding standard BRAF V600-targeted protocols). Blue bar sections indicate the proportion of patients receiving personalized recommendations for combined immune-checkpoint inhibitor (ICI) and kinase inhibitor regimens. Red bar sections indicate the proportion of patients without any systemic treatment recommendation or with recommendations restricted exclusively to clinical trial enrollment. CNA copy number alteration, EL evidence level, ICI immune-checkpoint inhibitor, MTB molecular tumor board, *n* patient count, NCT National Center for Tumor Diseases.

We manually clustered all genes that guided therapeutic recommendations within the MTB-FR. In addition to classical *BRAF* V600 and atypical *BRAF* variants, we identified 31 distinct potentially targetable oncoproteins. The most frequent oncogenic aberrations occurred in *NRAS* (44%)*, ATM* (17%)*, BRCA2* (15%)*, ERBB4* (13%), *ATRX* (12%), and *EGFR* (10%), whereas other molecular targets demonstrated frequencies below 10% (Fig. 4a). Alterations in *NRAS*, atypical *BRAF* mutations, *CDK4/6* pathway amplifications, and mutations in DDR-related genes most frequently prompted biomarker-guided therapeutic recommendations. Specifically, the most commonly recommended regimen was a combination of a CDK4/6 inhibitor (ribociclib or abemaciclib) and a MEK inhibitor (binimetinib), guided by an *NRAS* Q61 mutation with or without concurrent *CDK4/6* pathway amplification or *CDKN2A* deletion (*n* = 35). This was followed by the recommendation of CDK4/6 inhibitors alone (*n* = 14), PARP inhibitors (*n* = 13), or EZH2 inhibitors (*n* = 11) based on alterations in genes involved in the DDR pathway, such as *BAP1*, *RAD50*, and *ATM*. Furthermore, dabrafenib or sorafenib combined with the MEK inhibitor trametinib (*n* = 8) or a novel MEK/panRAF inhibitor (DRKS00015849) (*n* = 5) were repeatedly recommended based on pathogenic variants within the MAPK/RAF pathway^55^. Genetic aberrations guiding the final consensus MTB therapeutic recommendation are annotated with asterisks (*). Notably, whether an alteration translated into a clinical recommendation was ultimately determined by the interplay of the specific genetic event, concurrent mutations, and the patient’s clinical status (Figs. 4a, b and S4a). Table 1 provides an overview of the molecular targets identified in the MTB-FR cohort alongside their corresponding treatment recommendations and exemplary therapeutics.

**Table 1:** A translational matrix correlates actionable molecular alterations with targeted therapeutic recommendations

Signaling pathway	Gene	Specific alteration	ClinVar	REVEL score	NCT level	Recommended drug(s)	Supporting literature/clinical trials
MAPK/RAF							
	BRAF	p.G466E	n.k.	n.k.	m3	Sorafenib & Trametinib	PMID: 27523909, PMID: 28783719
	BRAF	p.F468S	n.k.	n.k.	m3	Sorafenib & Trametinib	Internal data (Brummer lab)
	BRAF	p.K601E	n.k.	n.k.	m1c	Dabrafenib & Trametinib	PMID: 30248172, PMID: 28344857
	BRAF	p.G593D	n.k.	0.914	m1c	Sorafenib & Trametinib	PMID: 39177935, PMID: 31580757
	BRAF	p.N581S	n.k.	n.k.	m1c	Dabrafenib & Trametinib	PMID: 36217799 and internal data (Brummer lab)
	BRAF	p.V276A	n.a.	0.869	n.k.	MEKi, panRAFi	Internal data (Brummer lab)
	BRAF	p.G469A	pathogenic	0.9	m1c	Dabrafenib & Trametinib	PMID: 32981611; PMID: 35869122
	BRAF	p.E501A	n.k.	n.k.	m3	MEKi, panRAFi	PMID: 32873792 and internal data (Brummer lab)
	BRAF	MKRN1::BRAF	n.a.	n.a.	m2b	panRAFi, Belvarafenib	PMID: 33953400
	BRAF	MACF1::BRAF	n.a.	n.a.	m1c	MEKi	PMID: 24345920, PMID: 26314551
	RAF1	p.I606M	n.k.	n.k.	m3	panRAFi, Naporafenib	PMID: 19727074; PMID: 31581174
	NF1	p.R440X, p. Q1822X	pathogenic	n.a.	m1b	MEKi	PMID: 31151904, PMID: 26936308
	NRAS	p.Q61K	conflicting interpretations	0.841	m1b	Ribociclib/Binimetinib	PMID: 35294522
	NRAS	p.Q61R	pathogenic	0.888	m1b	Ribociclib/Binimetinib	PMID: 35294522
	NRAS	p.Q12D	pathogenic/likely pathogenic	0.783	m1b	Ribociclib/Binimetinib	PMID: 35294522
	NRAS	p.Q61L	uncertain significance	0.897	m1b	Ribociclib/Binimetinib	PMID: 35294522
Cell cycle							
	CDK4	Amplifikation	n.a.	n.a.	m1b	CDK4/6i	PMID: 33770575
	CCND1	Amplifikation	n.a.	n.a.	m1b	CDK4/6i	PMID: 33770575, PMID: 27217383, PMID: 27542767
	CCND3	Amplifikation	n.a.	n.a.	m1b	CDK4/6i	PMID: 33770575, PMID: 27217383, PMID: 27542768
	CCNE	Amplifikation	n.a.	n.a.	m1b	CDK4/6i	PMID: 33770575, PMID: 27217383, PMID: 27542770
	CDKN2A	p.D84N	conflicting interpretations	0.67	m1b	CDK4/6i	PMID: 33770575, PMID: 27217383, PMID: 27542767
	CDKN2A	p.H83Y	uncertain significance	0.804	m1b	CDK4/6i	PMID: 33770575, PMID: 27217383, PMID: 27542767
	CDKN2A	p.P48L	n.k.	0.765	m1b	CDK4/6i	PMID: 33770575, PMID: 27217383, PMID: 27542767
	CDKN2A	p.R80X	pathogenic/likely pathogenic	0.48	m1b	CDK4/6i	PMID: 33770575, PMID: 27217383, PMID: 27542767
	CDKN2A/B	Deletion	n.a.	n.a.	m1b	CDK4/6i	PMID: 33770575, PMID: 27217383, PMID: 27542767
	PPP6C	p.H114Y	n.k.	n.k.	m3	Trametinib	PMID: 33789117
DNA damage response (DDR)							
	ATR	p.Q1533*	n.k.	n.k.	m3	WEEi	PMID: 32343890, PMID: 33119476
	ATM	p.P1378L	uncertain significance	0.546	m2a	PARPi	PMID: 32343890, PMID: 33119476
	ATM	p.Q355*	pathogenic	n.k.	m2a	PARPi	PMID: 32343890, PMID: 33119476
	ATM	p.M2531T	n.k.	0.196	m2a	PARPi	PMID: 32343890, PMID: 33119476
	BAP1	p.E642Dfs*5	n.k.	n.k.	m2b	PARPi	PMID: 34661178
	BAP1	p.R56Sfs*10	n.k.	n.k.	m2b	EZH2i, Tazemetostat	PMID: 35588752
	BAP1	splice	n.k.	n.k.	m2b	EZH2i, Tazemetostat	PMID: 35588752
	CHEK2	splice	conflicting interpretations	n.k.	m2a	PARPi	PMID: 32343890, PMID: 33119476
	ERCC4	p.D330Tfs*8	n.k.	n.k.	m3	PARPi	PMID: 30797591, PMID: 36110934
	XRCC2	p.F32Lfs*30	conflicting interpretations	n.k.	m3	PARPi	PMID: 30797591, PMID: 36110934
	BRCA2	p.A75P	benign	0.324	m2a	PARPi	PMID: 28578601, PMID: 31157963, PMID: 32771088
	BRCA2	p.V145del	conflicting interpretations	n.k.	m2a	PARPi	PMID: 28578601, PMID: 31157963, PMID: 32771088
	BRCA2	p.P3324L	uncertain significance	0.059	m2a	PARPi	PMID: 28578601, PMID: 31157963, PMID: 32771088
	RAD	p.V146fs	n.k.	n.k.	m2b	PARPi	PMID: 29635390
Receptor tyrosine kinases and growth factors							
	EGFR	p.E758K	n.k.	n.k.	m2a	EGFRi, Osimertinib	PMID:29151359
	ERBB4	p.E452K	likely pathogenic	0.559	m2b	ERBB4i, Lapatinib	PMID 23365119; ASCO 2018 abstract 6087
	FGFR2	Amplifikation	n.a.	n.a.	m2b	FGFRi, Futibatinib	PMID: 34551969
	FGFR1	SEMA6A::FGFR1	n.a.	n.a.	m2a	FGFRi, Pemigatinib	PMID: 38710951
	FGF	Amplifikation	n.a.	n.a.	m1b	FGFRi	PMID: 21976540
	KIT	p.N822Y	n.k.	0.895	m1b	Ripretinib, Regorafenib	PMID: 21642685, PMID: 35753087; PMID: 28487491
	KIT	p.D820Y	pathogenic	0.915	m1b	KITi, Ripretinib	PMID: 35753087
	KIT	Amplifikation	n.a.	n.a.	m1b	Imatinib	PMID 21642685
	MET	mRNA Überexpression	n.a.	n.a.	m1b	Amivantamab oder Crizotinib	PMID: 28950372
**Oncogenic intracellular signaling pathways**							
	PIK3CA	p.E542K	pathogenic	0.931	m1b	Alpelisib	PMID: 38261444; PMID: 35335966, PMID: 30237495
	PIK3CD	p.E992K	pathogenic	0.627	m3	AKTi	PMID: 35335966, PMID: 30237495
	PTEN	Deletion	incomplete	n.a.	m2a	AKTi, Capivasertib	PMID: 37256976
	GNAQ/11	p.Q209L	pathogenic/likely pathogenic	0.936	m1b	Darovasertib und Crizotinib	doi.org/10.1016/j.annonc.2023.09.2215
	JAK2	KIAA2026::JAK2	n.a.	n.a.	m2a	JAKi	PMID: 32279331, PMID: 26569516
	PTCH1	p.Q563X	pathogenic	n.k.	m2b	Hedgehogi, Vismodegib	doi.org/10.1200/JCO.2022.40.16_suppl.3010
	ACVR1	Amplifikation	n.a.	n.a.	m2a	Momelotinib	PMID: 36861402, PMID: 29263442, PMID: 32915978
Nuclear and epigenetic pathways							
	SMARCB1	p.Q377X	n.k.	n.k.	m2b	EZH2i, Tazemetostat	PMID: 37228094; PMID: 29650362, PMID: 33035459
	SMARCB1	p.D69Afs*27	n.k.	n.k.	m2b	EZH2i, Tazemetostat	PMID: 37228094; PMID: 29650362, PMID: 33035459
	PBRM1	p.R1185X	n.k.	n.k.	m2b	EZH2i, Tazemetostat	PMID: 37228094; PMID: 29650362, PMID: 33035459
	PBRM1	p.W1157X	n.a.	n.a.	m2b	EZH2i, Tazemetostat	PMID: 37228094; PMID: 29650362, PMID: 33035459
	EZH2	p.R690C	n.k.	0.88	m2b	EZH2i, Tazemetostat	PMID: 37228094; PMID: 29650362, PMID: 33035459
	SF3B1	p.R625H	likely pathogenic	0.875	m3	PARPi	PMID: 35027467; DOI: 10.1182/blood-2022-170574
	SF3B1	p.R625C	likely pathogenic	0.823	m2b	ATRi oder IRAK4i	PMID: 37954584, DOI: 10.1097/01.HS9.0000845916.12740.98
Cellular metabolism							
	IDH1	p.R132C	pathogenic/likely pathogenic	0.814	m2a	IDHi, Ivosidenib	PMID: 29860938
	MTAP	Deletion	complete	n.a.	m1c	PRMT5i	PMID: 39293516
	TROP-2	positive protein expression	n.a.	n.a.	m2a	Sacituzumab govitecan	PMID:32223649

The matrix categorizes identified signaling pathways and specific genomic variants alongside bioinformatic validation criteria, assigned precision oncology evidence levels, and corresponding clinical rationales.

*ClinVar* Clinical Variants database, *DDR* DNA damage response, *i* inhibitor, *n.k.* not known, *NCT* National Center for Tumor Diseases, *REVEL* Rare Exome Variant Ensemble Learner.

Treatment recommendations were primarily driven by molecular aberrations identified within the MTB-FR, supplemented by data from routine diagnostics. To classify an alteration as a potential target, variants were cross-referenced with curated precision oncology databases to confirm functional oncogenic relevance. Recommendations were assigned to nationally established National Center for Tumor Diseases (NCT) evidence levels and prioritized accordingly^56,57^, which directly parallel the European ESCAT tier framework^58^. We preferentially suggested therapeutic options supported by the strongest evidence level, ELm1 (functionally corresponding to ESCAT Tiers I and II; 62.9%; *n* = 66 out of 105 recommendations), which derived either from phase I/II case-control studies or retrospective cohorts (ELm1b, 57.1%; *n* = 60) or from case reports and small case series within the same tumor entity (ELm1c, 5.7%; *n* = 6).

Because the transferability of drug efficacy from other malignancies to melanoma remains a key challenge, molecular therapeutic options validated only in different entities were assigned a lower priority under evidence level ELm2 (paralleling ESCAT Tier III; 28.6%; *n* = 30 of 105 recommendations). These were predominantly derived from prospective studies or meta-analyses (ELm2a, 14.3%; *n* = 15), retrospective cohorts or case-control studies (ELm2b, 13.3%; *n* = 14), and from case reports and small case series (ELm2c, 0.95%; *n* = 1). In cases involving rare mutations with a compelling biological rationale, molecular therapies were recommended based on preclinical data according to evidence level ELm3 (paralleling ESCAT Tiers IV and V; 8.6%, *n* = 9 of 105 recommendations) (Fig. 4c).

When evaluated on an individual patient basis, molecular profiling revealed at least one potential druggable target beyond the typical *BRAF* V600 mutation in 83.8% of patients (*n* = 67) among those with completed diagnostics (*n* = 80). Specifically, we detected a single molecular target in 50.0% (*n* = 40), two targets in 25.0% (*n* = 20), three targets in 8.8% (*n* = 7), and four targets in 1.3% (*n* = 1) of these patients (Fig. 4d). The majority of our molecularly profiled cohort benefited from high-evidence genomic mapping: exactly 53 unique patients (66.2% of those with completed diagnostics) harbored at least one actionable alteration classified as NCT-ELm1 (ESCAT Tier I/II).

Personalized therapeutic strategies extending beyond genomic profiling included the combination of the ICI pembrolizumab with the multi-kinase inhibitor lenvatinib^59^, a recommendation guided by a *BRAF* V600 wild-type status and ICI failure. Mechanistically, the board’s rationale was to leverage the anti-angiogenic properties of lenvatinib to modulate the immunosuppressive tumor microenvironment, thereby re-sensitizing refractory sub-clones to concurrent anti-PD-1 checkpoint inhibition. In contrast, for patients harboring an actionable *BRAF* V600 mutation, the board proposed a triple-therapy regimen combining a BRAF inhibitor, a MEK inhibitor, and an ICI^60,61^. The pharmacological rationale was to overcome cross-resistance to prior sequential treatments by simultaneously co-targeting the hyperactivated MAPK pathway and restoring susceptibility to immune checkpoint blockade. Crucially, a clinical prerequisite for both combination protocols was the absence of prior severe or dose-limiting immune-related adverse events (grade 3–4 irAEs) during frontline checkpoint inhibition, ensuring appropriate patient safety. Collectively, these personalized immunotherapy strategies were recommended for 72.4% of initially registered patients (*n* = 71 out of 98; Fig. S4b).

Enrollment into clinical trials was suggested based on either molecular profiling or individual clinical sub-profiles independent of genomic alterations. Utilizing the German national registry for oncological trials (QuickQueck©) (https://www.quickqueck.de/home/), at least one clinical trial could be proposed for 67.3% of patients (*n* = 66 out of 98) (Fig. S4b).

Overall, within the total melanoma cohort, we recommended at least one personalized treatment option outside of clinical trials in 86.7% of patients, and at least one molecularly stratified option independent of classical *BRAF* V600 alterations in 68.4% of cases. The proportion of patients for whom a biomarker-stratified therapeutic option was identified was higher in the *BRAF* wild-type melanoma subgroup (77.6%) compared to *BRAF* V600 mutant melanoma (47.8%) and uveal melanoma (50.0%), a distribution primarily driven by the higher prevalence of *NRAS* aberrations in the *BRAF* wild-type population (Fig. 4e).

Importantly, other therapeutic orientations, such as localized radiotherapy or surgical interventions, were not within the structural scope of the MTB-FR. At our institution, multimodal local strategies are strictly evaluated and decided within specialized, organ-specific tumor boards, allowing the MTB-FR to focus exclusively on the evaluation of advanced systemic therapies and multi-omic target characterizations when conventional lines fail.

### Implementation and outcome

**Fig. 5:**
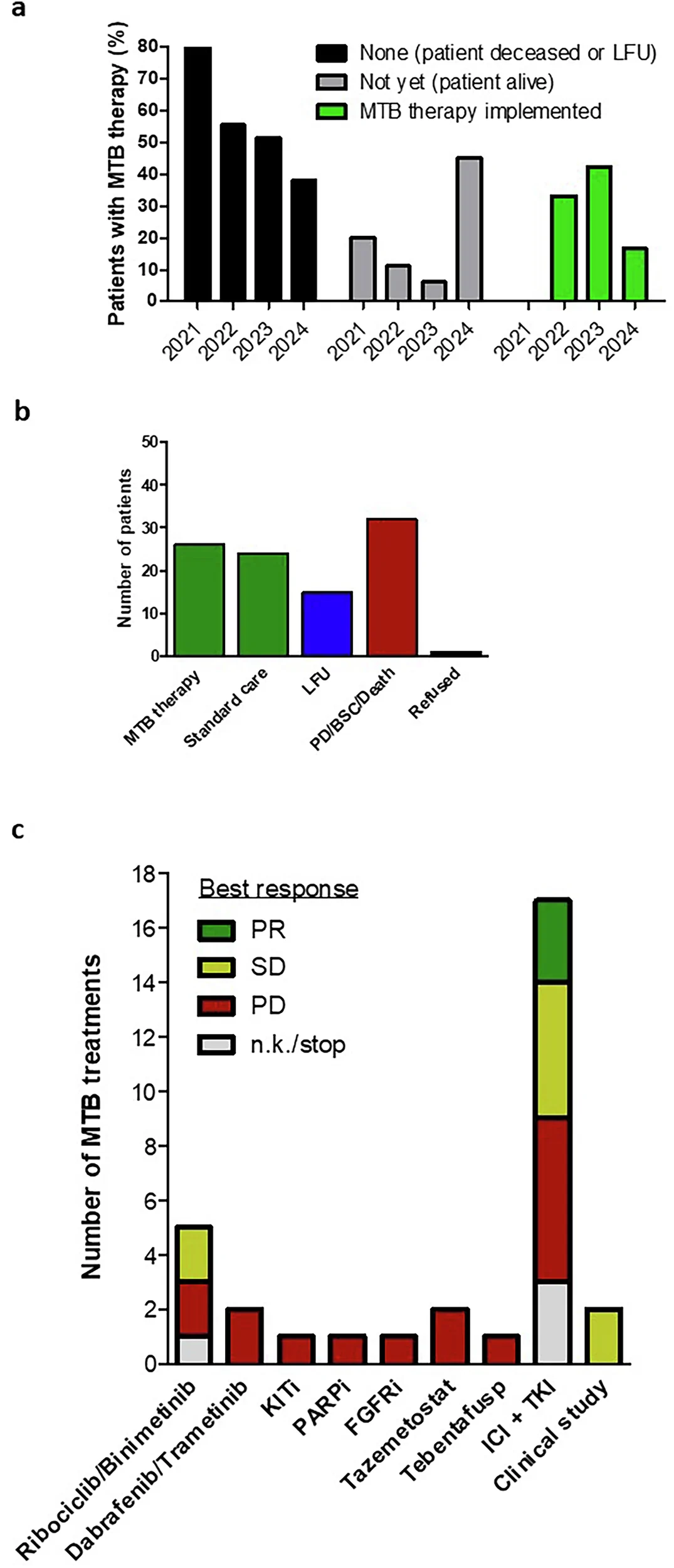
Cohort-level evaluation illustrates treatment implementation patterns and clinical outcomes. **a** Proportion of patients receiving MTB-guided therapies stratified by their respective calendar year of enrollment into the MTB-FR observational study. Categories comprise patients who died without therapeutic implementation or were lost to follow-up (none), patients who remain alive and are candidates for future implementation (not yet), and patients who successfully initiated one or two consecutive recommended MTB regimens (MTB therapy implemented). **b** Absolute number of patients implementing at least one recommended therapy line alongside a breakdown of specific clinical, logistical, or administrative barriers preventing implementation. **c** Bar graph displaying the best objective response to implemented MTB treatment recommendations, stratified by the absolute count of treated patients per therapeutic class. BSC best supportive care, CR complete response, irAE immune-related adverse event, LFU lost to follow-up, MTB molecular tumor board, n.k. not known, PD progressive disease, PR partial response, RECIST Response Evaluation Criteria in Solid Tumors, SD stable disease.

By the end of the observation period, 27 out of 80 patients with completed molecular diagnostics had received treatment according to the MTB-FR recommendations, corresponding to a therapy implementation rate of 33.8%. A total of 32 MTB-guided lines were administered, as five patients received two consecutive recommended regimens. Between 2021 and 2024, the proportion of patients who died without receiving an MTB-recommended therapy steadily decreased from 80.0 to 38.1%. Several patients, particularly those registered in 2024 with shorter follow-up intervals at the data cut-off, remain alive and are candidates for future MTB therapy implementation (Fig. 5a). This reduction in pre-treatment mortality coincided with the incremental workflow optimization and accumulating institutional experience over time. Clinicians increasingly referred high-risk individuals at an earlier, pre-emptive stage of disease failure, while parallel streamlining of genomic diagnostics and off-label cost-reimbursement applications was associated with reduced logistical delays before therapeutic initiation.

Reasons contributing to the non-implementation of MTB-FR recommendations across the remaining cohort included (1) continuation of standard-of-care therapy or treatment cessation due to ongoing adequate clinical response (CR, PR, or SD; *n* = 24), (2) loss to follow-up (LFU; *n* = 15), (3) transition to best supportive care (BSC) due to rapid clinical deterioration, poor performance status, or documented death prior to treatment initiation (*n* = 32), and (4) refusal of cost reimbursement by health insurance providers (*n* = 1) (Fig. 5b). The cohort of patients continuing standard therapy (*n* = 24) reflects our proactive registration workflow. These individuals either stabilized after anticipated progression or responded to standard bridging regimens during the diagnostic turnaround window, leaving the MTB recommendation as a future fallback option. In one patient case, insurance coverage was initially denied for the recommended combination of ribociclib and binimetinib. However, this individual previously received treatment access through enrollment in an MTB-FR-recommended clinical trial, and subsequently, insurance approval was secured for an alternative off-label combination of pembrolizumab and lenvatinib.

Personalized treatment strategies were implemented across three primary modalities (Fig. 5c): combined ICI and kinase inhibitor regimens in 17 patients (17.3% of the total cohort), enrollment into molecularly guided clinical trials in two patients (2.0%), and targeted biomarker-driven therapies in 13 patients (13.3%). These molecularly stratified options included ribociclib plus binimetinib (*n* = 5), dabrafenib plus trametinib (*n* = 2), tazemetostat (*n* = 2), or single-agent regimens utilizing a KIT inhibitor (*n* = 1), PARP inhibitor (*n* = 1), FGFR inhibitor (*n* = 1), or tebentafusp (*n* = 1). Efficacy outcomes varied across these subgroups. For patients receiving the combined ICI and kinase inhibitor protocols, a subset (*n* = 7 out of 13 evaluable) achieved SD (*n* = 4) or objective partial responses (*n* = 3), while a minority required treatment discontinuation due to dose-limiting toxicities (*n* = 2). Patients enrolled in clinical trials achieved SD as their best response. Among patients receiving ribociclib and binimetinib, disease stabilization and progression were distributed equally (*n* = 2 evaluable each), whereas the remaining molecularly-stratified single-agent therapies predominantly resulted in progressive disease (Fig. 5c).

**Fig. 6:**
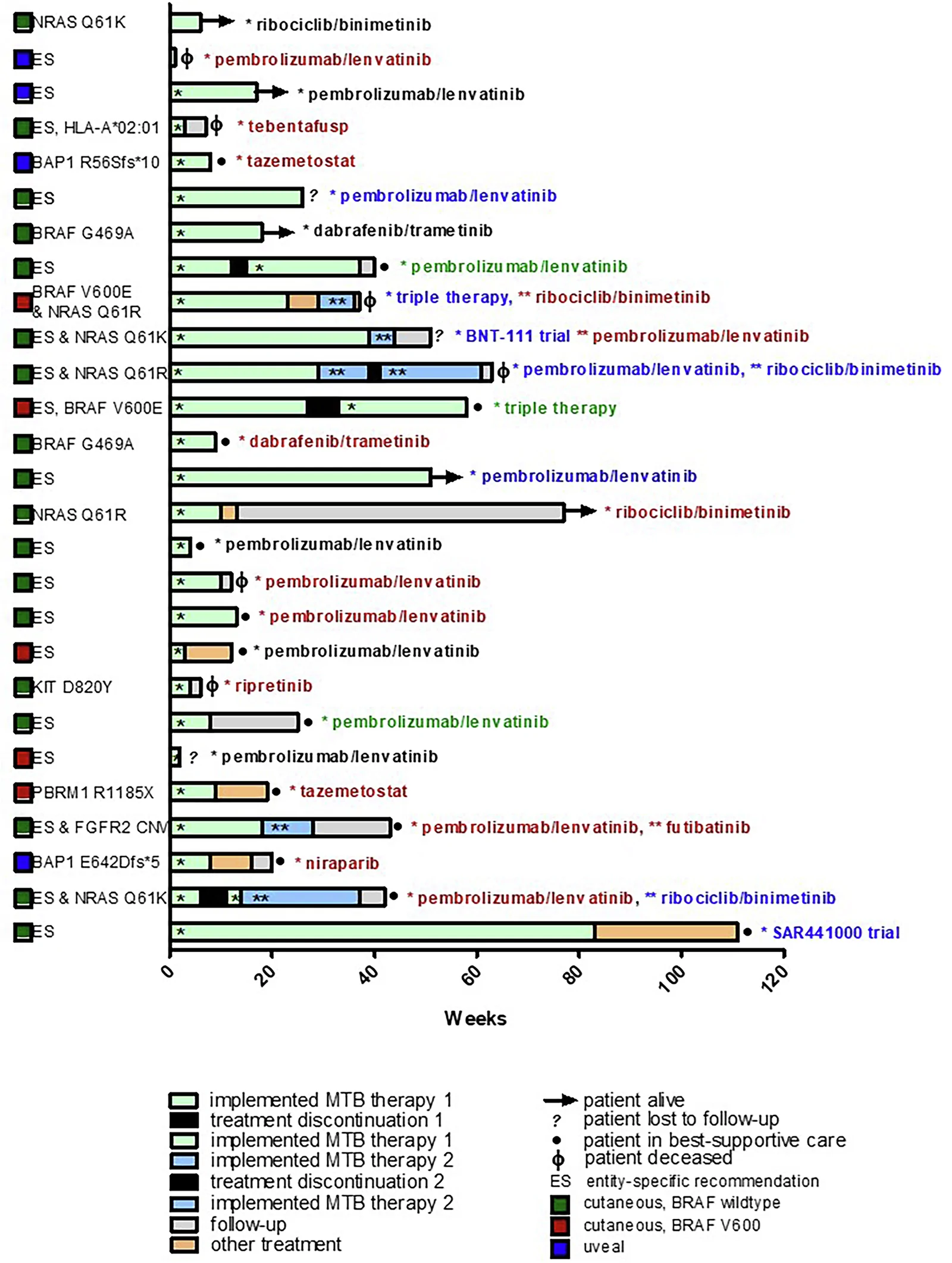
Chronological patient tracking captures longitudinal treatment courses and clinical outcomes. Modified swimmer plot delineating individual longitudinal treatment courses, toxicity-related interruptions, and survival follow-up periods for all treated patients within the melanoma cohort. Melanoma biological subtypes are color-coded along the y-axis, along with the specific genetic or molecular alteration that justified the therapeutic strategy, and the treatment duration in weeks is displayed along the x-axis. Green and blue horizontal bars represent the duration of the first (implemented MTB therapy 1) and second (implemented MTB therapy 2) lines of MTB-recommended treatment, respectively. Black horizontal bars indicate temporary treatment interruptions (e.g., due to dose-limiting toxicities), orange bars denote subsequent conventional non-MTB therapies, and gray bars illustrate follow-up intervals without active systemic treatment. Actionable target drugs are specified via annotations, with colors indicating the best objective response achieved per regimen according to RECIST v1.1 (green indicates partial response [PR], blue indicates stable disease [SD], red indicates progressive disease [PD], and black indicates response unknown or unevaluated). Ongoing treatment or survival at the data cut-off is indicated by terminal arrows. Patients lost to follow-up are designated by question marks, transitions to best supportive care are marked by black dots, and documented patient death is indicated by a dagger symbol. BSC best supportive care, CR complete response, irAE immune-related adverse event, LFU lost to follow-up, MTB molecular tumor board, PD progressive disease, PR partial response, RECIST Response Evaluation Criteria in Solid Tumors, SD stable disease.

Individual patient longitudinal data for all 27 individuals who underwent MTB-guided treatment are depicted in a modified swimmer plot (Fig. 6). Within this treated cohort, 18 patients (66.7%) were diagnosed with cutaneous *BRAF* V600 wild-type melanoma, five patients (18.5%) with cutaneous *BRAF* V600 mutant melanoma, and four patients (14.8%) with uveal melanoma. Excluding the five patients with ongoing treatment at the data cut-off, the mean duration of the first MTB-guided therapy line was 18.2 weeks (range: 1–83 weeks). Five of the 27 patients (18.5%) transitioned to a second MTB-recommended regimen, with a mean treatment duration of 15.0 weeks (range: 5–30 weeks). Regarding pharmacological dosing, all recommended systemic regimens were initially proposed at standard regulatory starting doses. However, to manage toxicities in this heavily pretreated cohort, subsequent adaptations of the therapeutic regimen were common, with 37.0% of patients requiring at least one formal dose reduction, mostly for lenvatinib. Consequently, toxicity-related management required temporary treatment interruptions exceeding 1 week in four patients (12.9%), and permanent discontinuation was necessary in three cases (9.7%). Six patients (22.2%) received subsequent conventional therapies after progressing on their MTB-guided regimen. Excluding patients who were still alive at the data cut-off, the mean OS following the initiation of the first MTB-guided therapy was 21.8 weeks. Ultimately, across the entire treated population, the overall disease control rate (DCR) following implementation of the recommended therapy was 42.9%, and the overall response rate (ORR) was 10.7% (Fig. 1a).

## Discussion

Despite extensive therapeutic advances, *BRAF* V600 mutations remain the sole oncogenic alterations with regulatory approval for targeted therapy in melanoma. MTBs aim to bridge this critical gap by translating comprehensive multi-omic profiling into personalized, real-world treatment strategies when standard guideline-based algorithms fail. The vast majority of melanoma patients with advanced disease eventually experience progression or treatment-limiting toxicities under frontline ICI^7,62^. In this refractory salvage setting, the MTB-FR serves as an exploratory precision oncology platform, uncovering alternative, biomarker-guided therapeutic avenues beyond established standards of care. This prospective observational study highlights the clinical feasibility of an institutional molecular tumor board (MTB-FR) leveraging multi-omic and orthogonal proteomic workflows for heavily pretreated, refractory advanced cutaneous and uveal melanoma patients.

Our cohort exhibited a lower proportion of *BRAF*-mutant cutaneous melanoma (26% versus 74% *BRAF* non-V600 disease, excluding uveal melanoma) compared to unselected historical cohorts^63–67^. This divergence may reflect the availability of standard frontline BRAF/MEK inhibition, which potentially retains clinical progressors within standard protocols before the utilization of molecular profiling. With regard to the 41% prevalence of brain metastases, our collective is consistent with the aggressive landscape characteristic of advanced melanoma populations^68,69^. However, this critical clinical manifestation frequently shows limited responsiveness to systemic therapies, including individualized MTB-recommended regimens, and thus remains an ongoing therapeutic challenge^70^.

An important observation of our genomic mapping is the identification of atypical non-V600 *BRAF* aberrations in 13.4% of patients previously managed under standard frontline protocols as *BRAF* wild-type disease, a proportion slightly higher than that observed in unselected cohorts^43,44^. Because *BRAF* alterations may confer a high risk of disease relapse, these patients potentially accumulate within MTB cohorts and could benefit from systematic evaluation for personalized targeted treatment options. This enrichment illustrates that routine diagnostic sequencing may occasionally overlook complex alternative drivers, which might be intercepted with targeted multi-kinase or pan-RAF inhibitor regimens. This is particularly relevant when mutations or gene fusion events affect *BRAF* outside of the routinely screened hotspot exons 11 and 15. Furthermore, our cohort captures potential evolutionary selection pressure induced by targeted therapies, as evidenced by two patients progressing under standard BRAF/MEK inhibition who acquired secondary *NRAS* Q61 variants while maintaining their initial *BRAF* V600E or V600K driver mutations. The emergence of these dual-positive, multi-clonal sub-phenotypes highlights a fundamental escape mechanism via reactivation of the MAPK axis, supporting the rationale for vertical pathway blockade or alternative combinations in the post-resistance setting.

Methodologically, this study addresses an important precision oncology bottleneck: the frequent discrepancy between genomic CNVs and actual protein abundance. By incorporating high-resolution, antibody-independent LC-MS-based proteomics, we confirmed proteomic target upregulation, such as directly quantifying CDK4 overabundance, providing additional biological support for CDK4/6 inhibition. However, our pipeline suggests that bulk LC-MS-based proteomics may serve as an unbiased scouting tool rather than a standalone decision metric. Because bulk analyses cannot localize protein expressions to malignant cells or differentiate them from peritumoral stroma, orthogonal, spatially resolved IHC validation remains highly valuable to verify tumor-cell-specific receptor compartmentalization. The necessity of this dual-platform framework is clearly illustrated when evaluating novel ADC structures in our cohort. Our selective screening revealed that established ADC targets, including Nectin-4, HER2, FRα, and Claudin-18.2, are generally uncommon at the protein level in advanced melanoma. This low IHC detection rate aligns with independent proteomic reference data^54^ and current literature suggesting that markers like Nectin-4 may only emerge in specific resistant subphenotypes^71^. Conversely, the identification of a single patient with actionable TROP-2 expression highlights a novel observation, as data on TROP-2 landscapes in melanoma and clinical responses to sacituzumab govitecan remain highly limited^72^. While current data are insufficient to draw definitive conclusions regarding broader ADC efficacy in melanoma, these findings support the feasibility of our workflow, where explorative LC-MS proteomics expands the target discovery space, and IHC adjudicates the in situ expression required for clinical application. Looking ahead, while targeted tumor-only panel sequencing paired with proteomics offers a pragmatic framework to mitigate genomic noise, future workflows could benefit from matched tumor-normal WES.

Beyond classical *BRAF* and *NRAS* hotspot mutations, comprehensive profiling revealed at least one additional potential druggable target in 83.8% of successfully sequenced cases (*n* = 67 out of 80). These biomarker-guided recommendations were most frequently driven by *NRAS* mutations, atypical *BRAF* variants, CDK4/6 pathway amplifications, and alterations in DDR-related genes. Notably, the majority of these therapeutic recommendations were supported by intermediate-to-low levels of clinical evidence, specifically falling into the NCT categories ELm1b, ELm2, and ELm3 (functionally corresponding to ESCAT Tiers II to IV). While high-level actionable drivers (such as standard *BRAF* V600 alterations) are routinely managed via standard protocols outside the board, patients referred to the MTB-FR frequently presented with rare variants or complex co-alterations lacking prospective clinical trial data. Recommending off-label regimens based on retrospective data, cross-entity transferability (ELm2), or preclinical modeling (ELm3) reflects a common strategy in a heavily pretreated population with exhausted conventional options. This reliance on lower-level evidence highlights a persistent challenge in precision oncology: the widening gap between rapid multi-omic discovery and the availability of high-evidence clinical trials for rare tumor profiles. Ultimately, our real-world data illustrates the potential benefit of international basket and umbrella trials to evaluate these non-hotspot alterations within the specific context of melanoma biology.

Our observed therapy implementation rate of 33.8% at the data cut-off represents a dynamic baseline rather than a final outcome. Longitudinal tracking revealed a decline in pre-treatment mortality over time (from 80 to 38.1%), suggesting a potential institutional learning curve. This shift indicates that moving multi-omic profiling from a late-stage salvage attempt to an early, preemptive screening layer during anticipated frontline failure could help mitigate logistical and clinical delays before therapeutic initiation. Due to our institutional strategy toward early registration during proactive disease management, 45.2% of patients enrolled in 2024 still exhibit ongoing responses to standard frontline therapy. Given this preemptive sequencing strategy, these individuals may potentially be considered for MTB-recommended regimens upon future progression. Regarding specific treatment modalities, combined ICI and kinase inhibitor regimens were implemented in 17.3% of cases, molecularly stratified targeted agents in 13.3%, and clinical trial enrollment in 2.0%. Notably, a discrepancy emerged between the high rate of clinical trial recommendations (71.4%) and this low actual enrollment (2.0%). This real-world attrition likely reflects screening failures where specific inclusion or exclusion criteria could not be met, insufficient tissue biomaterial for trial-specific biobanking, or logistical constraints, including patient non-eligibility or refusal due to the travel required to reach external study sites.

Although disease progression remained a dominant factor in this heavily pretreated, refractory collective, our observations are comparable to published benchmark data. For patients receiving combined ICI and multi-kinase or MAPK pathway inhibition, the observed clinical courses align with the benchmark phase II LEAP-004 trial (pembrolizumab plus lenvatinib) after frontline anti-PD-1 failure, which reported an ORR of 21.4%^59^. Similarly, recent multicenter trials evaluating anti-PD-1 rescue combined with BRAF/MEK inhibition post-ICI progression reported an ORR of 35.4% and a DCR of 47.9%^73^. Direct comparisons to treatment-naïve cohorts (e.g., IMPemBra or KEYNOTE-022 trials^60,61^) are inherently precluded. Conversely, molecularly stratified therapies yielded more modest outcomes within our cohort; patients receiving the ribociclib and binimetinib combination achieved a DCR of 50% (two SDs, two progressive diseases), consistent with the phase Ib/II trial framework in *NRAS*-mutant melanoma, where an ORR of 19.5 to 32.5% was associated with co-occurring cell-cycle aberrations. Other single-agent targeted interventions (e.g., KIT, PARP, or FGFR inhibitors) were restricted to single individuals and resulted in progressive disease. Given the very limited size of these specific subgroups, no definitive conclusions can be drawn, and larger cohorts are required to further evaluate the clinical role of these specific precision strategies.

We acknowledge certain limitations in our study, primarily the relatively small sample size of the uveal melanoma subgroup (8%), which reflects the rarity of this entity but limits definitive statistical assertions regarding its distinct *GNAQ*/*GNA11*-driven biology. A further limitation of this study is the absence of a randomized control arm, meaning our data primarily illustrate the technical and operational feasibility of our multi-omic pipeline rather than definitive clinical efficacy. Any comparisons regarding survival or response durations remain strictly indirect and benchmarked against historical literature. Given the small sample size of our treated subcohort (n = 27) and the high clinical heterogeneity of advanced melanoma progressors, cross-trial comparisons must be interpreted with caution. Consequently, while our pipeline appears suitable for genomically mapping refractory patients and generating consensus recommendations within a clinically actionable timeframe, randomized prospective trials remain essential to evaluate whether these individualized strategies translate into a reproducible survival benefit over standard subsequent therapies. Furthermore, the therapeutic outcomes observed within our treated collective may be influenced by survival bias. Because comprehensive multi-omic sequencing and off-label cost-reimbursement applications require an operational window of several weeks, patients with rapidly accelerating disease kinetics may experience clinical deterioration or death before treatment initiation, thereby inherently selecting a subcohort with more favorable tumor kinetics. However, this potential survival bias appeared to be progressively mitigated over time as clinicians increasingly referred patients preemptively during anticipated frontline therapy failure. This heavily pretreated and refractory state may also explain the observed low ORR of 10.7%. In patients who have already experienced multi-line failure on standard-of-care immune-checkpoint blockade and targeted therapies, the tumor architecture is frequently characterized by multi-clonal resistance networks. In this advanced setting, achieving disease stabilization, as reflected in our DCR of 42.9%, highlights the potential to achieve disease control within a highly aggressive malignancy, even if objective tumor shrinkage remains rare.

In conclusion, our data illustrate the clinical and operational feasibility of integrating comprehensive multi-omic profiling into the pipeline of a tertiary care medical center. While these real-world findings do not provide definitive proof of clinical efficacy, they indicate that it is operationally viable to cross-analyze complex genomic and proteomic landscapes, formulate personalized recommendations in 67.3% of melanoma cases (reaching 85.7% when incorporating personalized immunotherapy combinations), and navigate the logistics of off-label drug access. Integrating protein-based diagnostics, such as high-resolution mass spectrometry and targeted IHC, alongside classical sequencing can expand the target discovery space, enhancing the likelihood of identifying alternative, tailored pathways when standard therapies have failed. Ultimately, translating this demonstrated diagnostic feasibility into reproducible clinical benefits highlights the necessity for subsequent prospective, randomized evaluation within well-defined biomarker-stratified patient populations.

## Methods

### Study design and patient cohort

The MTB-FR is a single-center, multidisciplinary platform comprising oncologists, physicians from various specialties, pathologists, molecular biologists, and medical bioinformaticians, with the aim of recommending biomarker-guided, personalized therapy options for cancer patients in a real-world setting^35^. In brief, the treating physician decides to register and initially presents the patient’s case at the MTB-FR. This first interdisciplinary discussion defines the molecular profiling strategy, including the selection of the specific gene sequencing modality. Upon completion of the molecular characterization, a second and, if required, third interdisciplinary discussion is held to establish the final consensus MTB recommendation, which encompasses both personalized and molecularly stratified treatment options. The operational timeline involves a 2-to-6-week window for advanced molecular tissue testing. Following report availability, cases are reviewed at the next biweekly board meeting, resulting in a cumulative 4-to-8-week interval from initial diagnostic request to the formal consensus recommendation. The subsequent time to clinical treatment initiation is highly variable, ranging from a few weeks to months. This wide span reflects a proactive profiling strategy, wherein high-risk patients undergo sequencing during anticipated frontline therapy failure to preemptively secure a personalized salvage strategy.

The MTB-FR observational study was approved by the Independent Ethics Committee of the University of Freiburg Medical Faculty (Approval No. 369/19) and conducted in accordance with the Declaration of Helsinki. All included patients provided written informed consent.

The analysis evaluated a subcohort of 98 patients diagnosed with advanced cutaneous or uveal melanoma who were enrolled in the prospective MTB-FR observational study (DRKS00025847) between January 2021 and December 2024. Inclusion criteria covered patients with cutaneous or uveal melanoma at AJCC 8th edition^36^ stage III or IV, aged at least 18 years, treated at the University Medical Center Freiburg, Germany, and exhibiting a documented or anticipated exhaustion of standard-of-care therapy options as determined by the treating physician. The data cut-off for clinical outcome assessment was September 1st, 2025. Clinical and radiographical response to implemented therapies was systematically evaluated by certified radiologists using cross-sectional imaging (computed tomography [CT] or magnetic resonance imaging [MRI]) according to the Response Evaluation Criteria in Solid Tumors (RECIST) version 1.1 guidelines.

### Molecular tumor sequencing and protein biomarker analyses

During the initial MTB-FR discussion, the most suitable tumor material was selected as either formalin-fixed paraffin-embedded (FFPE) or fresh-frozen (FF) tissue. After morphological microdissection of tumor cells, automated tumor DNA and RNA extraction were performed using the Maxwell® RSC DNA and RNA FFPE Kits on a Maxwell RSC 48 instrument (Promega, Madison, WI, USA). Extracted DNA and RNA concentrations were quantified using a Qubit fluorometer (Thermo Fisher Scientific, Waltham, MA, USA) and a TapeStation system (Agilent Technologies, Santa Clara, CA, USA). Material quality was determined via the DNA Integrity Number (DIN) and RNA Integrity Number (RIN), respectively. DNA from the normal control samples used for WES was extracted from plasma-depleted whole blood (PDWB) using the QIAamp DNA Mini Kit (QIAGEN, Hilden, Germany) according to the manufacturer’s instructions.

Targeted NGS using the TSO500 panel (Illumina, San Diego, CA, USA), which evaluates 523 genes for SNVs, TMB, microsatellite instability [MSI], and CNVs in DNA, alongside 55 genes for RNA fusions and splice variants, was performed at the Institute of Surgical Pathology. In selected cases, a separate large RNA fusion panel (507 genes; Illumina), immunohistochemistry (IHC), and ISH were conducted within accredited diagnostic workflows. The mass spectrometry (MS)-based proteomic screening was executed at the same institute using a non-accredited, exploratory protocol. WES was performed at the NGS Core Facility of the German Cancer Research Center (DKFZ, Heidelberg, Germany). For IHC, the following specific antibodies were utilized: PD-L1 (Clone SP263, Ventana Medical Systems, Tucson, AZ, USA), TROP-2 (Clone EPR20043, Abcam, Cambridge, UK), Nectin-4 (Clone EPR15613-68, Abcam), Folate Receptor alpha (Clone BN3.2, Novocastra, Leica Biosystems, Wetzlar, Germany), HER2 (Clone 4B5, Ventana Medical Systems), Claudin-18.2 (Clone 43-14A, Ventana Medical Systems), CDK4 (Clone DCS-35, Invitrogen, Carlsbad, CA, USA), and MITF (Clone 34CAS, Leica Biosystems).

Following microscope-guided microdissection, proteins were extracted, prepared, proteolytically digested, and analyzed qualitatively and quantitatively by LC-MS using an Evosep One HPLC system (Evosep Biosystems, Odense, Denmark) coupled to a timsTOF fleX mass spectrometer (Bruker Daltonics, Bremen, Germany) in data-independent acquisition mode, as previously described^74^. MS-based proteomic data were analyzed against the European Bioinformatics Institute (EBI) protein database (January 2025 release; including the Claudin-18.2 isoform). Standardized quality criteria were applied, including a 1% false discovery rate considering unique peptides only, followed by log2-transformation of protein abundance values.

For WES, libraries of tumor and germline DNA were prepared using the SureSelect XT and SureSelect Human All Exon V5+UTR or V6+UTR target enrichment kits (Agilent Technologies). Sequencing was executed on Illumina platform-based instruments (HiSeq 4000, NovaSeq 6000, or NextSeq 550) at the DKFZ/DKTK-MASTER program (Heidelberg, Germany). Post-sequencing quality control and adapter trimming were performed using FastQC^75^ and Trimmomatic^76^. Somatic variant calling was conducted via Mutect2 and VarScan2, followed by false-positive filtering according to GATK best practices^76–80^. Tumor-somatic variants were distinguished from germline variants by cross-referencing tumor sequencing files with matched constitutional controls. Only non-synonymous SNVs and small insertions/deletions (indels) exhibiting a VAF > 5% and with a population frequency of <0.1% (minor allele frequency, MAF) within the Genome Aggregation Database (gnomAD) were reported^81^. Actionable variants were annotated according to ClinVar, InterVar, OncoKB, and established cancer hotspots, with functional predictions derived from the dbNSFP database^82–88^.

Separate RNA-sequencing (RNA-Seq) for structural rearrangement detection as performed in specific cases using the SMARTer Ultra Low Input RNA Kit v4 (Takara Bio, Shiga, Japan) and sequenced on the aforementioned Illumina platforms. High-throughput RNA fusions were inferred utilizing FusionCatcher^89^ and Arriba^90^. Data visualization was executed within the R framework using the Bioconductor package maftools^91,92^, Python (library matplotlib)^93^, as well as a customized^94–96^ version of cBioPortal^97^.

### Personalized treatment recommendations

During the initial interdisciplinary board discussion, preliminary personalized recommendations are formulated based on preexisting routine diagnostic data. These encompass non-molecular treatment modalities or molecularly stratified therapies, such as inclusion in clinical trials or ICI and kinase inhibitor regimens. The search for appropriate clinical studies is systematically conducted utilizing the national registry for oncological studies, QuickQueck© (https://www.quickqueck.de/home/). Upon completion of comprehensive molecular profiling, subsequent interdisciplinary discussions are held to establish definitive biomarker-guided treatment options or molecularly driven clinical trial recommendations. Molecular alterations are classified and prioritized according to the nationally established NCT evidence level framework^35,56,82^. This tier-based system categorizes recommendations into: evidence within the same tumor entity (ELm1), evidence in a different tumor entity (ELm2), preclinical evidence (ELm3), or an underlying biological rationale (ELm4). For clinical actionability, levels ELm1 and ELm2 are further sub-stratified based on the strength of the underlying clinical data: prospective trials or meta-analyses (A), retrospective cohorts or case-control studies (B), and single case reports or exceptional responders (C).

### Implementation, response criteria and outcome

For the MTB-FR melanoma cohort analysis, all clinical, genomic, proteomic, and clinical outcome data were retrospectively collected and evaluated. Cutaneous melanoma was categorized into *BRAF* V600 wild-type versus *BRAF* V600-mutant subgroups based on routine molecular diagnostics performed at initial diagnosis. The clinical implementation of the final MTB-FR recommendation was decided by the treating physician after obtaining informed patient consent. The therapy implementation rate was calculated as the number of patients receiving at least one MTB-guided treatment line divided by the total number of patients with successfully completed molecular profiling.

Treatment duration was calculated in weeks from the initiation of the recommended MTB therapy to the date of documented treatment cessation, death from any cause, or the last follow-up. A full week of treatment was credited if therapy was administered for at least four out of 7 consecutive days. A treatment interruption was defined as any continuous suspension of therapy lasting 7 days or longer. Radiographical tumor response was assessed via routine clinical cross-sectional imaging (CT and/or MRI scans), and the best objective response to the MTB-guided treatment was retrospectively evaluated. The DCR was defined as the proportion of patients achieving a complete response (CR), partial response (PR), or SD as their best objective response to the recommended MTB therapy. The ORR was defined as the proportion of patients achieving either a CR or PR. OS was calculated from the date of the first MTB-guided therapy initiation to the date of death from any cause, with alive patients censored at the date of their last follow-up.

### Statistical analyses

Continuous variables were presented using median and standard error, mean or median and 25%, 50%, and 75% percentiles as indicated. Statistical computations and data visualizations were executed utilizing R (version 4.5.2) and GraphPad Prism (version 5.03), Boston, MA, USA.

## Supplementary information

Supplementary File

## Data Availability

The main data supporting the findings of this study are available within the article and its Supplementary Information files. Owing to ethical and legal restrictions related to the dissemination of human germline sequencing information stipulated in the informed consent forms of the MTB-FR observational study, the raw sequencing datasets cannot be deposited in public repositories. However, de-identified genomic variants (e.g., annotated somatic mutation lists) and normalized proteomic expression data are available from the corresponding author upon reasonable request. Access to pseudonymized raw sequencing data for validation purposes can be requested from the corresponding author, subject to approval by the local Ethics Committee and the execution of a formal Data Transfer Agreement (DTA).

## Code availability

No proprietary custom software code or programming algorithms were developed for the data processing or clinical analyses within this study. Somatic variant calling and quality control were executed using established public pipelines, including FastQC, Trimmomatic, Mutect2, and VarScan2. Structural rearrangements from RNA sequencing were inferred using the open-source tools FusionCatcher and Arriba. Genomic alterations were compiled and visualized using a customized institutional instance of the cBioPortal platform, the R package maftools and ggplot2, and the Python package matplotlib. General data aggregation, clinical cohort tracking, and the generation of downstream figures and statistical charts were executed utilizing Microsoft Excel and GraphPad Prism (version 5.03). All software tools utilized are standard, commercially or publicly available applications, and further inquiries regarding the bioinformatic architecture may be directed to the corresponding author.
