# Supplementary material for: Precision oncology in advanced melanoma: molecular landscapes and personalized therapy recommendations from the Molecular Tumor Board Freiburg
Source: NPJ Precis Oncol. 2026 Oct 21;10:400. doi: 10.1038/s41698-026-01763-w (PMC13687070; doi:10.1038/s41698-026-01763-w)
Supplement: Supplementary file 1 [file 41698_2026_1763_MOESM1_ESM.pdf]

Supplementary File, Lowinus et al. 2026

Title

Precision oncology in advanced melanoma: Molecular landscapes and personalized therapy recommendations from the Molecular Tumor Board Freiburg

Authors and Affiliations

Lowinus T.[1,2], Schipperges V.[3], Metzger P.[3], Brummer T.[2,4,5], Matysiak U.[6], Dopfer E.-P.[2,6], Hess M.E.[3], Fahrner M.[5,6], Pauli T.[3], Blaumeiser A.[3], Becker H.[1,2,4], Kuehn J.[1,2], Rassner M.[1,2], Falkenstein J.[1], Bertemes H.[1], Bovio P.[1], Pinter N.[6], Cysar S.[2,6], Lehr S.[7], Wäsch, R.[1], Gräßel, L.[1,2,4,8], Eyerich K.[7], Schultheis A.M.[2,4,6], Schilling O.[2,4,6], Lassmann S.[2,4,6], Werner M.[2,4,6], Illert A.L.[1,8,9,10], Duyster J.[1,2,4], Zeiser R.[1,2,4], Miething C.C.[1,2], Boerries M.[2,3,4], Meiss F.[7], Rafei D.[7#]

[1] Department of Medicine I - Medical Centre - University of Freiburg, Faculty of Medicine, University of Freiburg, Freiburg, Germany.

[2] Comprehensive Cancer Center Freiburg, Medical Center-University of Freiburg, Faculty of Medicine, University of Freiburg, Freiburg, Germany

[3] Institute of Medical Bioinformatics and Systems Medicine (IBSM), Medical Center – University of Freiburg, Faculty of Medicine, University of Freiburg, Freiburg, Germany

[4] German Cancer Consortium (DKTK), Partner Site Freiburg, a partnership between DKFZ and Medical Center - University of Freiburg, Freiburg, Germany

[5] Institute of Molecular Medicine and Cell Research (IMMZ), Faculty of Medicine, University of Freiburg

[6] Institute for Surgical Pathology, Medical Center-University of Freiburg, Faculty of Medicine, University of Freiburg, Freiburg, Germany.

[7] Department of Dermatology and Venereology – Medical Centre – University of Freiburg, Faculty of Medicine, University of Freiburg, Freiburg, Germany.

[8] Department of Hematology and Medical Oncology, University Medical Center Göttingen, Göttingen, Germany

[9] Department of Medicine III, Faculty of Medicine, Klinikum Rechts der Isar, Technical University Munich (TUM), 80333 Munich, Germany.

[10] Center of Personalized Medicine, Technical University of Munich (TUM), University Hospital, Technical University of Munich, 80333 Munich, Germany.

[#] Corresponding author: david.rafei-shamsabadi@uniklinik-freiburg.de

Supplementary Fig. 1. Baseline characteristics document the demographics and pretreatment clinical profiles of the cohort.

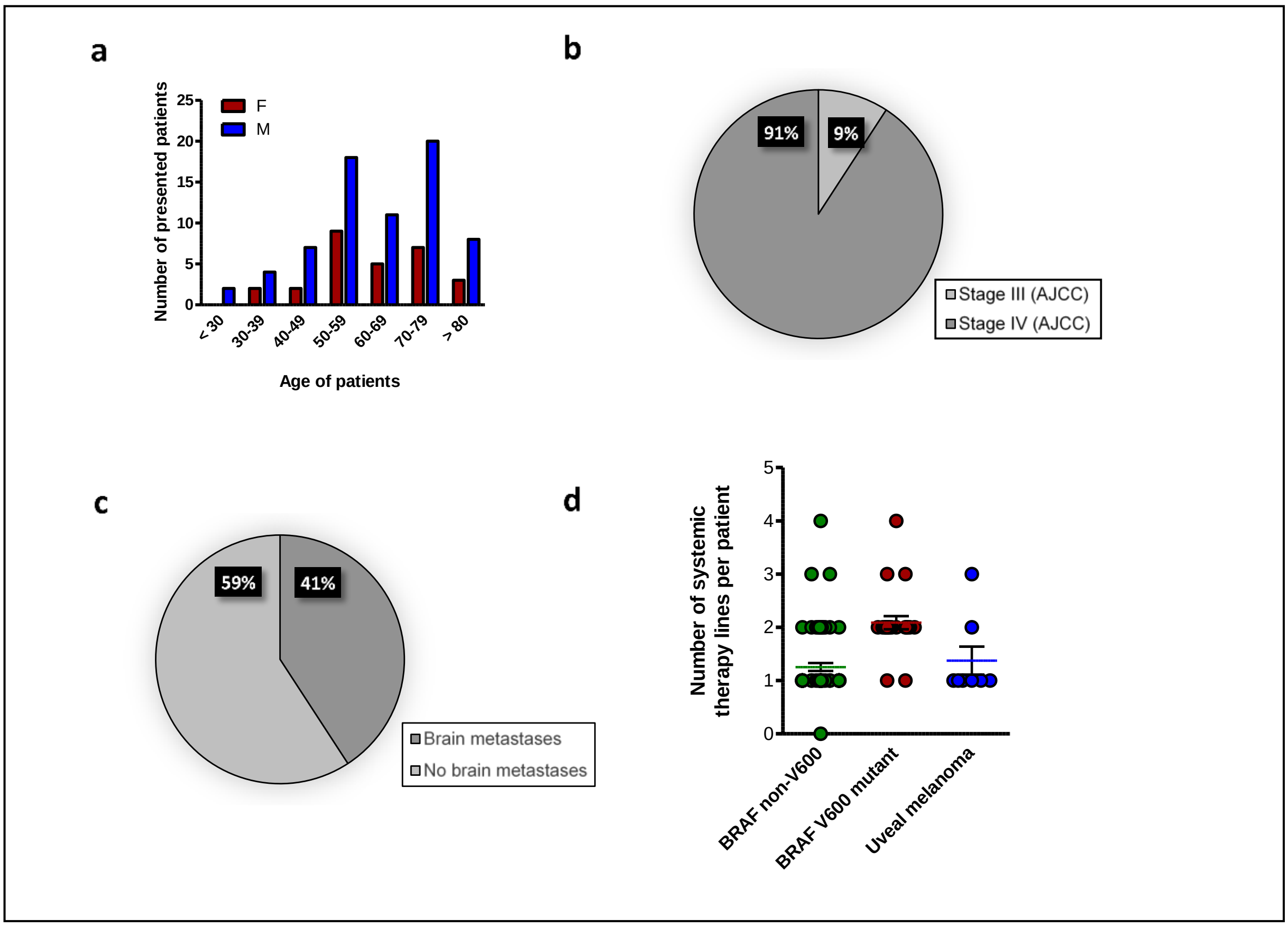

a Age group and gender distribution of melanoma patients enrolled in the MTB-FR observational study. **b** Pie chart presenting the distribution of stage III and stage IV AJCC tumor stages within the cohort. **c** Pie chart displaying the percentage of melanoma patients presenting with confirmed brain metastases within the cohort. **d** Number of prior systemic therapy lines per patient prior to enrollment in the MTB-FR observational study, stratified by disease biology into BRAF wild-type (non-V600-mutant), BRAF V600-mutant, and uveal melanoma. Each plot symbol represents an individual patient, where horizontal lines indicate the mean and error bars depict the standard error of the mean (SEM).

Abbreviations: AJCC, American Joint Committee on Cancer; BRAF wild-type, non-V600-mutant; MTB-FR, Molecular Tumor Board Freiburg; n, patient count; SEM, standard error of the mean.

Supplementary Fig. 2. Exploratory mutational analyses map signature exposures, variant distributions, and copy number variations.

a

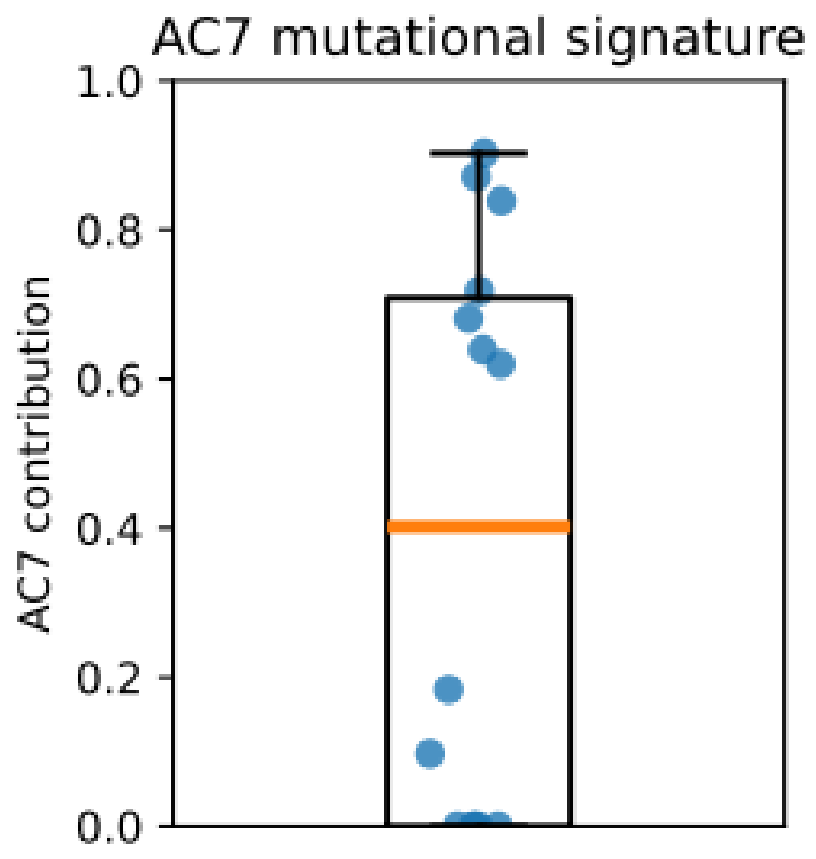

b

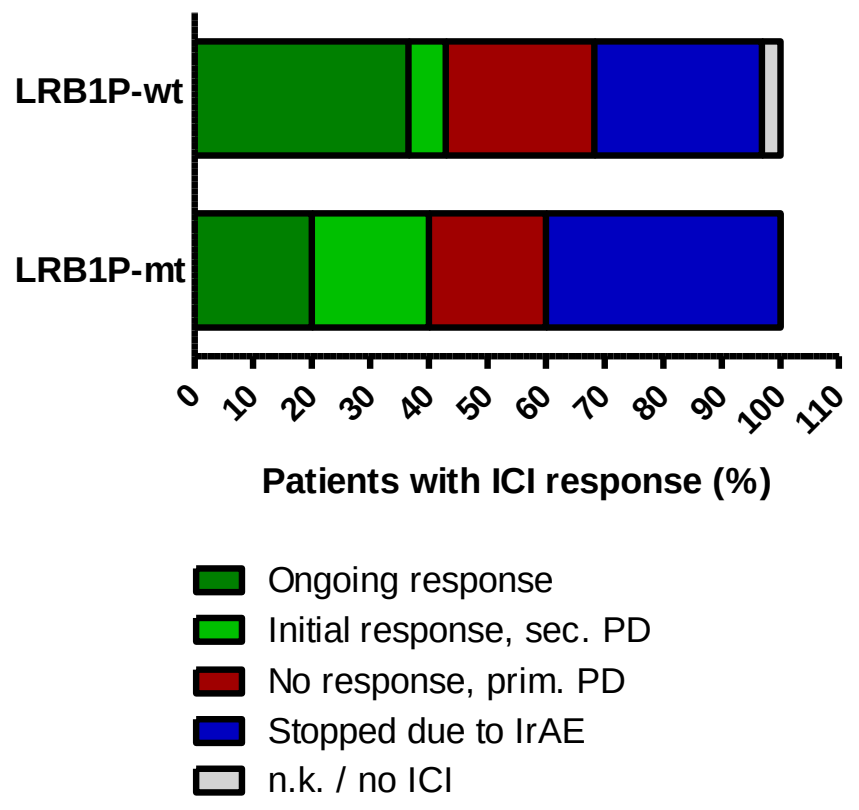

c

Atypical *BRAF* aberration	Category
V276A	class II
F468S	class II
G469A	class II
G469A	class II
K601E	class II
G466E	class III
E501A	class III
N581S	class III
G593D	class III
MACF1::BRAF	fusion
MKRN1::BRAF	fusion

d

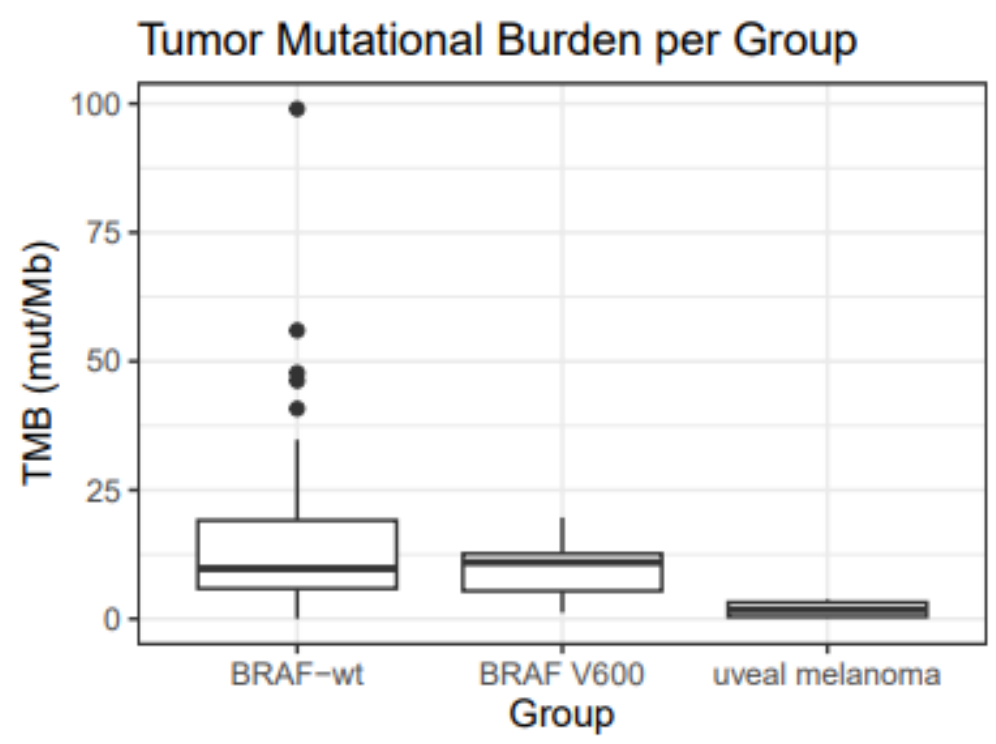

e

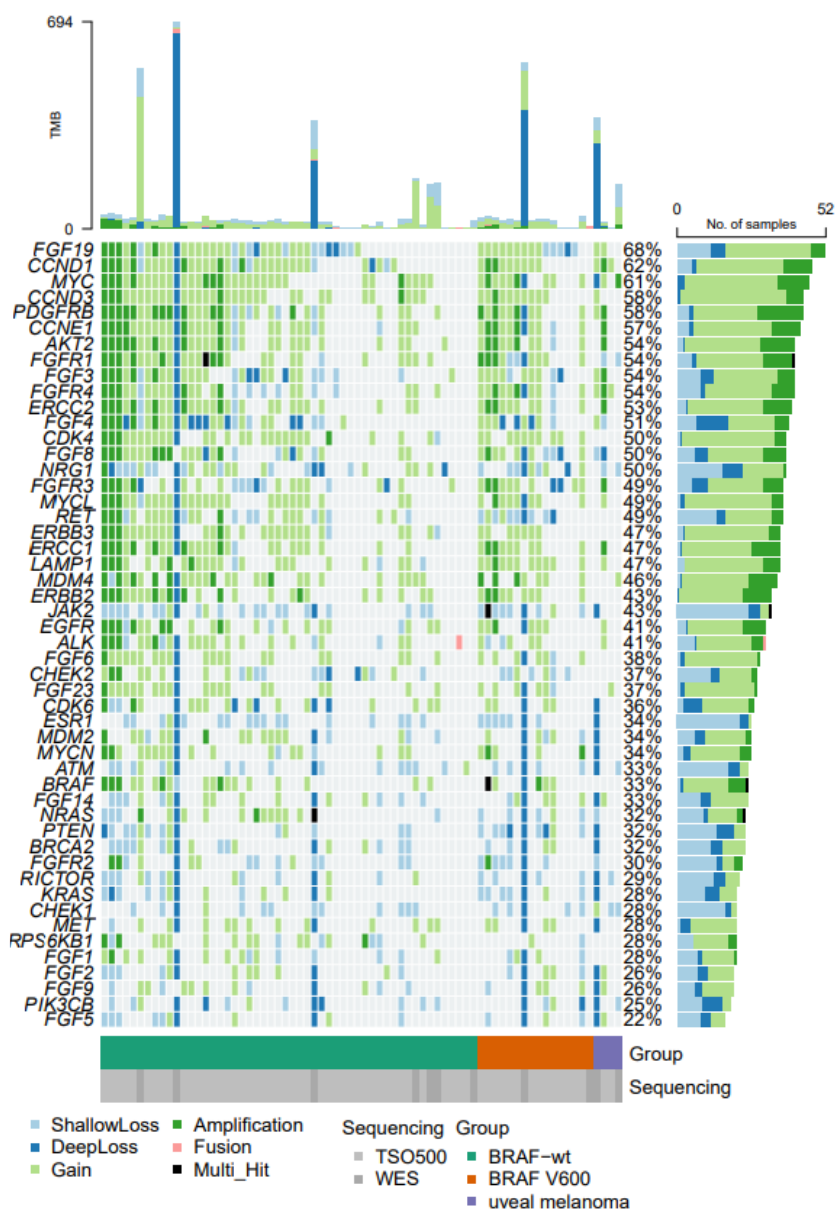

a Deconvolution of the classical COSMIC ultraviolet (UV) light mutational signature (SBS7) within the whole-exome sequencing (WES) subcohort (n = 14), depicting individual sample signature exposure weights. **b** Evaluation of ICI treatment responses stratified by *LRP1B* status in patients with *LRP1B* wild-type versus *LRP1B*-mutant tumors. Response categories comprise ongoing clinical benefit at the data cut-off, initial ICI response followed by secondary drug resistance, primary refractory disease, treatment discontinuation due to severe (grade 3–4) immune-related adverse events (irAEs), and ICI-naïve patients. **c** Supplementary Table 1 listing atypical, non-V600 *BRAF* alterations detected within the MTB-FR melanoma cohort via WES or TSO500 sequencing, including their respective functional classifications based on established literature[1,2]. **d** Box plots displaying individual TMB values (mutations per megabase; mut/Mb) across the overall collective, grouped by molecular subtypes into, *BRAF* non-V600 ("wild-type"), *BRAF* V600-mutant, and uveal melanoma. The horizontal internal line indicates the median, the box bounds represent the interquartile range (IQR; 25th and 75th percentiles), and the vertical whiskers depict the overall range. **e** OncoPrint matrix visualizing copy number variations (CNVs) across the molecularly profiled cohort genotyped melanoma patients via WES or the TSO500 assay, grouped into three cohorts: *BRAF* non-V600 ("wild-type") cutaneous melanoma, *BRAF* V600-mutant cutaneous melanoma, and uveal melanoma. Each column represents an individual tumor specimen, and each row corresponds to a specific gene. The percentage of altered cases per gene is depicted as a bar graph on the right. Individual sequencing modalities, clinical subgroups, and tumor mutational burden (TMB) values are aligned vertically.

Abbreviations: AC7, Alexandrov-COSMIC mutational signature 7 (ultraviolet light exposure); CNV, copy number variation; COSMIC, Catalogue of Somatic Mutations in Cancer; CR, complete response; ICI, immune checkpoint inhibitor; IQR, interquartile range; irAE, immune-related adverse event; mt, mutant; MTB-FR, Molecular Tumor Board Freiburg; mut/Mb, mutations per megabase; n, patient count; n.k., not known; PD, progressive disease; SBS7, single-base substitution signature 7; TMB, tumor mutational burden; TSO500, TruSight Oncology 500; UV, ultraviolet; WES, whole-exome sequencing; wt, wild-type.

Supplementary Fig. 3. Additional protein profiles document immunohistochemical target distribution and case-specific proteomic benchmarks.

a

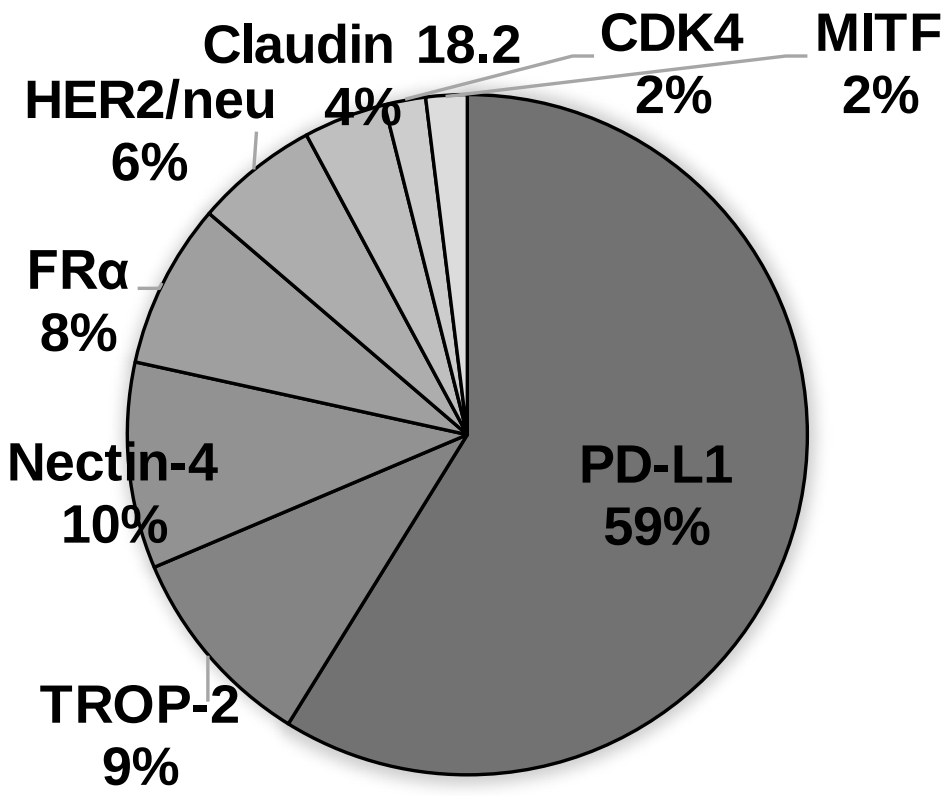

b

Protein	Z-Score	Distribution in reference cohort	Coverage in reference cohort n = 63
ADC targets			
NECTIN4	-0,87		8 %
TACSTD2 (TROP-2)	0,99		25 %
ERBB3 (HER3)	-0,63		95 %
Not identified: ERBB2 (HER2), CLDN18.2, FOLR1 (FRα), CD19, CD22 (SIGLEC2), CD33 (SIGLEC3), CD79B, TNFRSF8, TNFRSF17 (BCMA), F3 (Tissue Factor)			

c

Protein	Z-Score	Distribution in reference cohort	Coverage in reference cohort n = 63
Other therapeutical targets			
PTEN	-1,03		73 %
STING1	0,99		100 %
CDK4	2,44		100 %
MTAP	0,62		98 %
NF1	-0,49		97 %
PARP1	2,09		100 %
EFNB1	-1,54		43 %
Not identified: CD274 (PD-L1), PDCD1 (PD-1), CDK6, EGFR, MET, PDGFA/B, PDGFRA/B, VEGFA/B, DLL3, FOLH1 (PSMA), CTLA4, LAG3, MGMT, PARP2, FGFR1, FGFR2, FGFR3, FGFR4, FGF1-14, FGF16-23			

a Distribution of performed immunohistochemistry (IHC) stainings across the cohort, stratified by the indicated protein targets. **b, c** High-resolution mass spectrometry-based proteomic profiles for two illustrative melanoma cases from the cohort. For each target protein, panels display: (i) the patient's individual z-score (normalized intensity); (ii) the corresponding z-score distribution within a published melanoma reference cohort, represented as box plots where the center line indicates the median, the box limits represent the interquartile range (IQR), and the whiskers depict the 5th–95th percentiles; and (iii) the proportion of reference samples in which the specific target protein was confidently quantified ("coverage"). The blue cross denotes the exact value of the respective MTB-FR patient.

Abbreviations: ADC, antibody–drug conjugate; IHC, immunohistochemistry; IQR, interquartile range; MS, mass spectrometry; MTB-FR, Molecular Tumor Board Freiburg.

Supplementary Fig. 4: Stratified treatment recommendations outline biomarker-guided and personalized therapeutic frequencies.

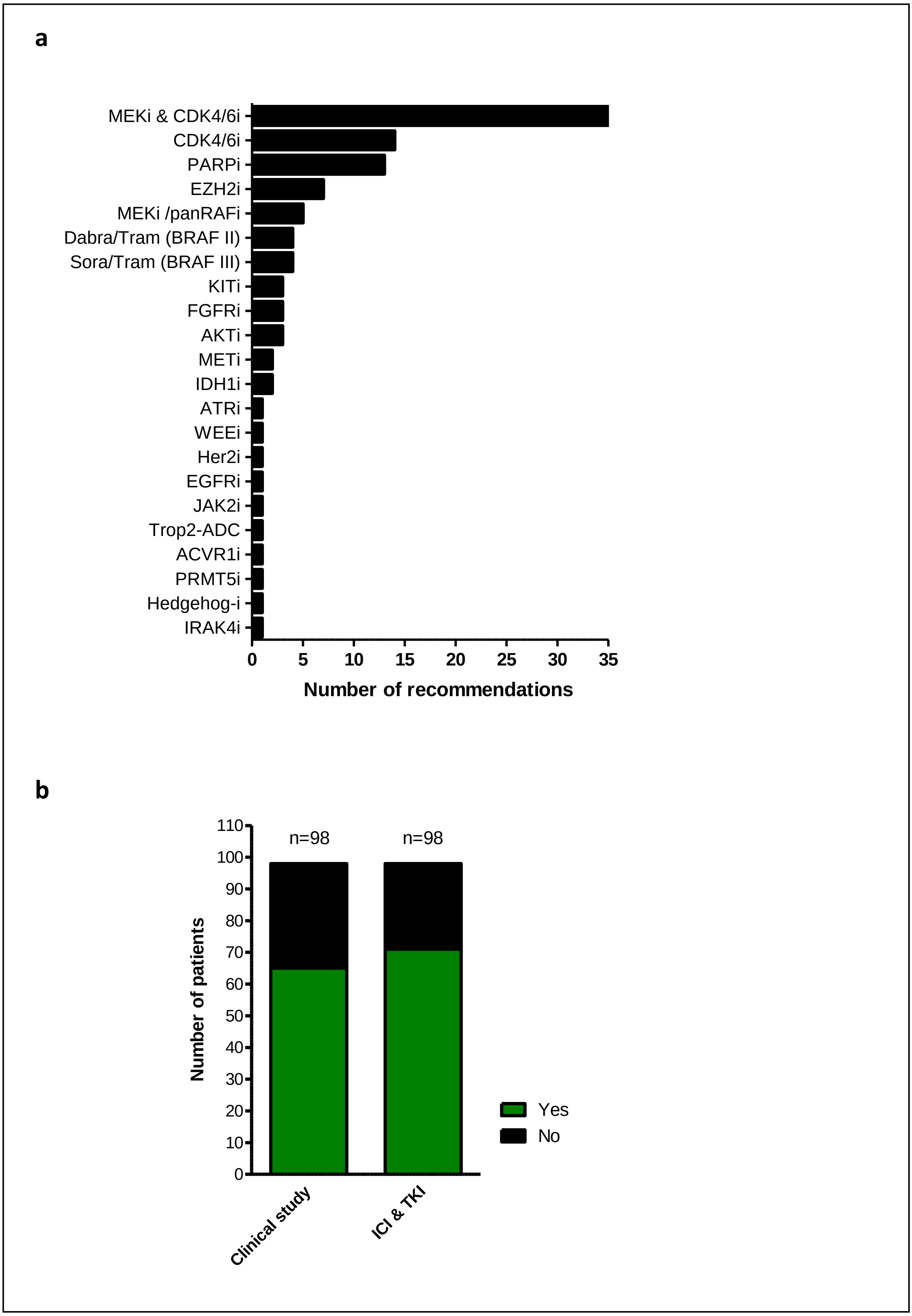

a Absolute frequency of specific molecularly stratified treatment recommendations formulated across the overall melanoma cohort, categorized by precise inhibitor classes. **b** Proportional distribution of patients receiving personalized MTB-FR recommendations designated either for inclusion into active clinical trials or for combined immune-checkpoint inhibitor (ICI) and kinase inhibitor regimens.

Abbreviations: I, inhibitor; ICI, immune-checkpoint inhibitor; MTB-FR, Molecular Tumor Board Freiburg.

Supplementary References.

1 Dankner, M., Rose, A. A. N., Rajkumar, S., Siegel, P. M. & Watson, I. R. Classifying BRAF alterations in cancer: new rational therapeutic strategies for actionable mutations. *Oncogene* **37**, 3183–3199 (2018). https://doi.org/10.1038/s41388-018-0171-x

2 Menzer, C. *et al.* Targeted Therapy in Advanced Melanoma With Rare BRAF Mutations. *J Clin Oncol* **37**, 3142–3151 (2019). https://doi.org/10.1200/JCO.19.00489
